# Supplementary material for: New dicationic DABCO-based ionic liquids: a scalable metal-free one-pot synthesis of bis-2-amino-5-arylidenethiazol-4-ones
Source: R Soc Open Sci. 2019 Jul 24;6(7):190997. doi: 10.1098/rsos.190997 (PMC6689602; doi:10.1098/rsos.190997)
Supplement: Supporting Information [file rsos190997supp1.pdf]

Electronic Supporting Information

New Di-cationic DABCO-based Ionic Liquids: A Scalable Metal-Free One-pot Synthesis of Bis-2-amino-5-arylidenethiazol-4-ones

Wael Abdelgayed Ahmed Arafa,*[a,b] and Asmaa Kamal Mourad[b]

[a]Chemistry Department, College of Science, Jouf University, P.O. Box 2014, Sakaka, Aljouf, Kingdom of Saudi Arabia.

[b]Chemistry Department, Faculty of Science, Fayoum University P.O. Box 63514, Fayoum City, Egypt.

E-mail: waa00@fayoum.edu.eg

Index

No.	Content	Page No.
1.	Copies of NMR spectra	S2-S29
2.	HRMS and ^{1}H NMR of (2-hydroxy-3-(hydroxymethyl)-5-methylphenyl)(piperidin-1-yl)methanone	S30, S31
3.	Experimental details for **9a-m** synthesis	S31-S36
4.	SEM and TEM of TiO_2 and ZnO NPs	S36-S39
5.	Green Metrics Calculations	S40
6.	References	S40

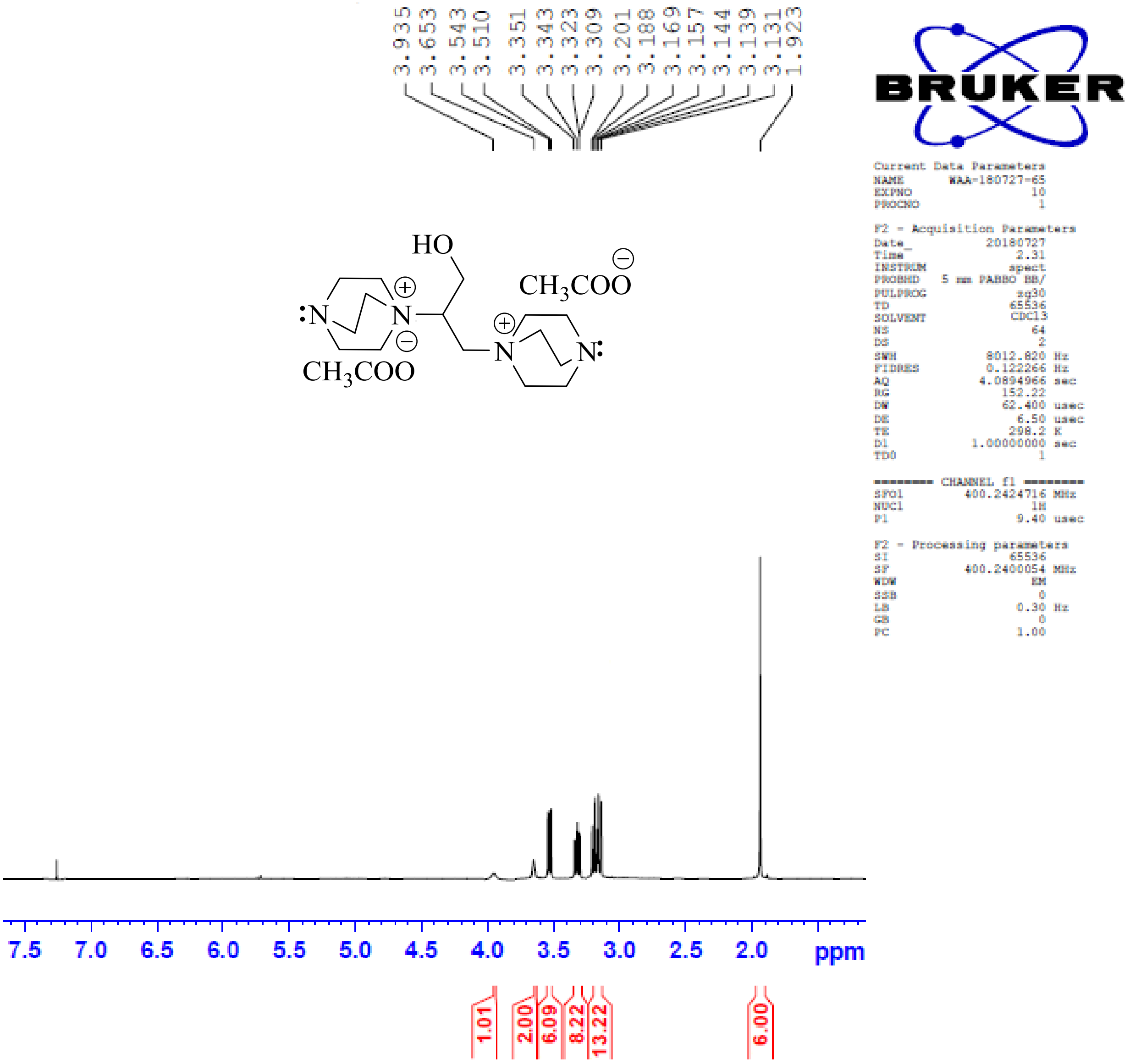

^{1}H NMR ($CDCl_3$): **3a**

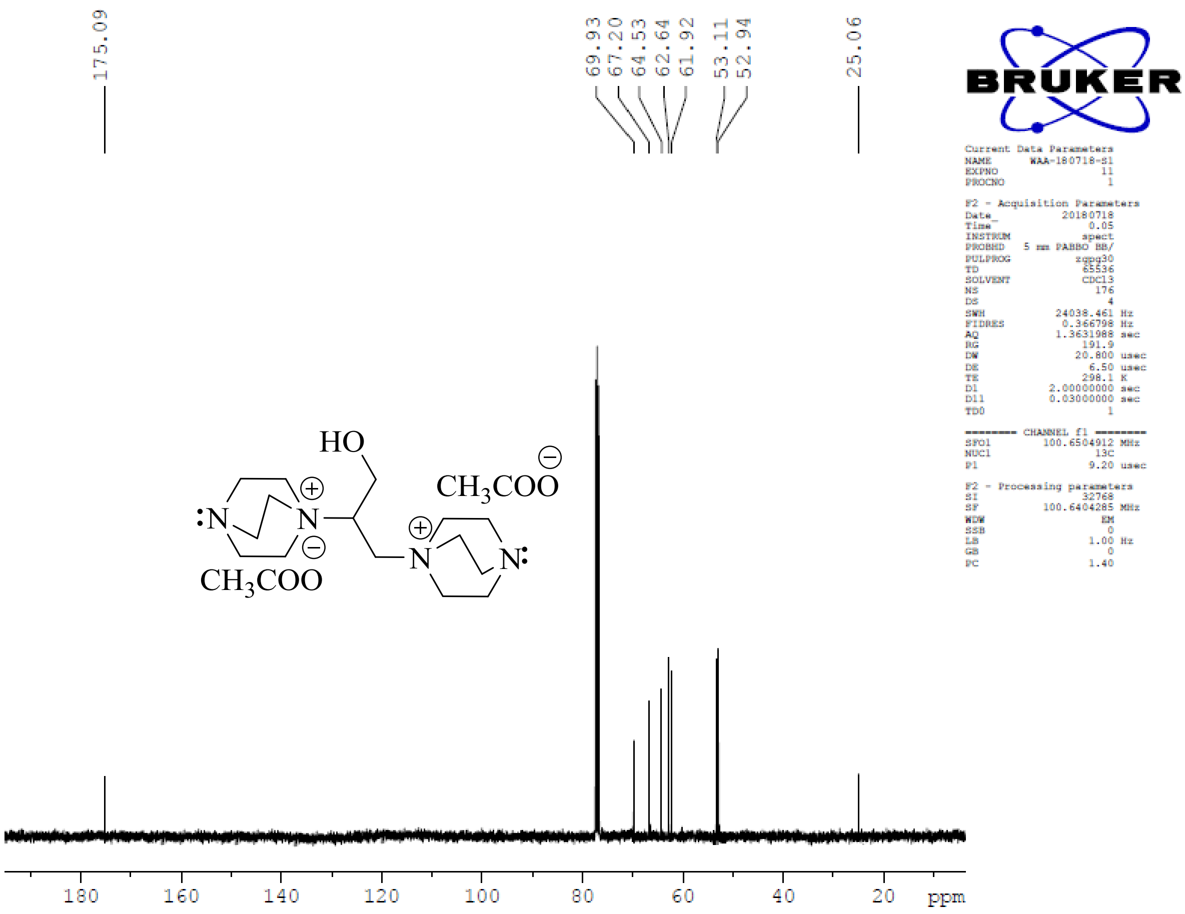

^{13}C NMR ($CDCl_3$): **3a**

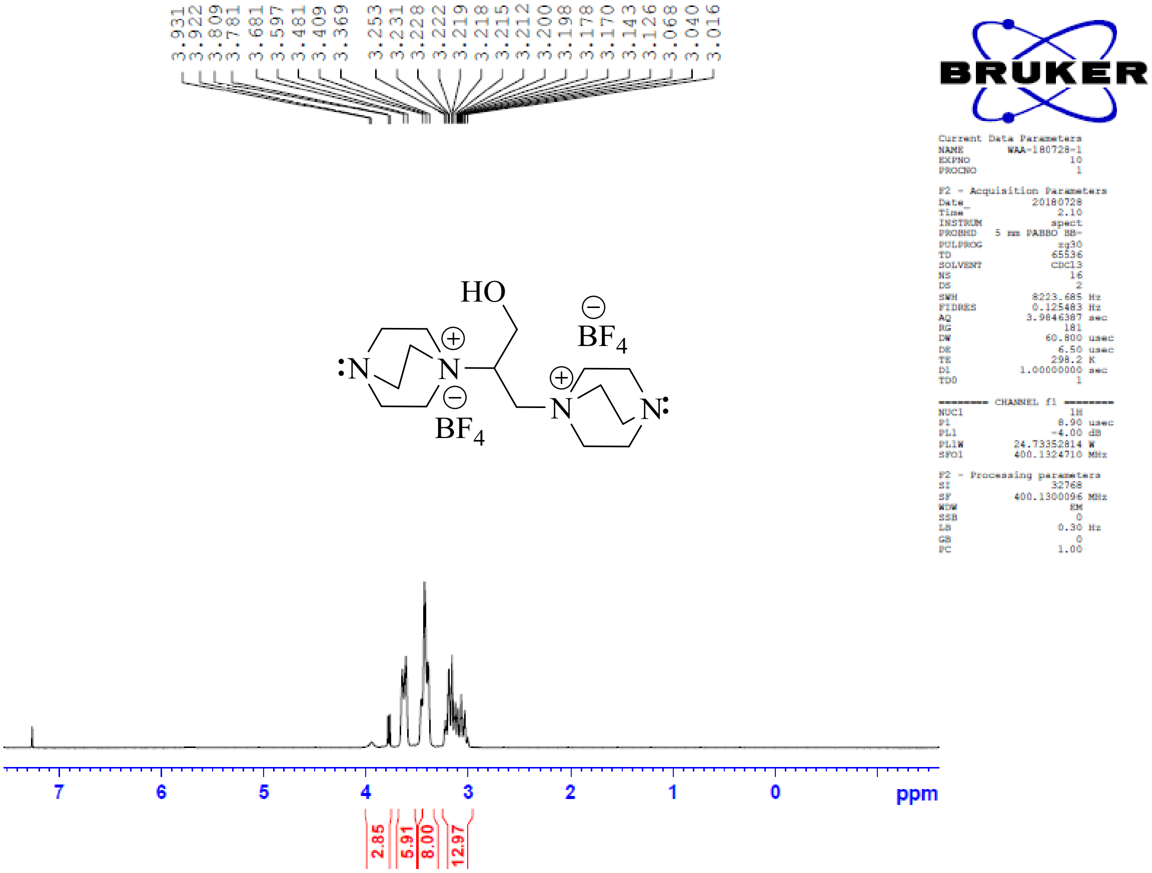

^1H NMR ($CDCl_3$): **3b**

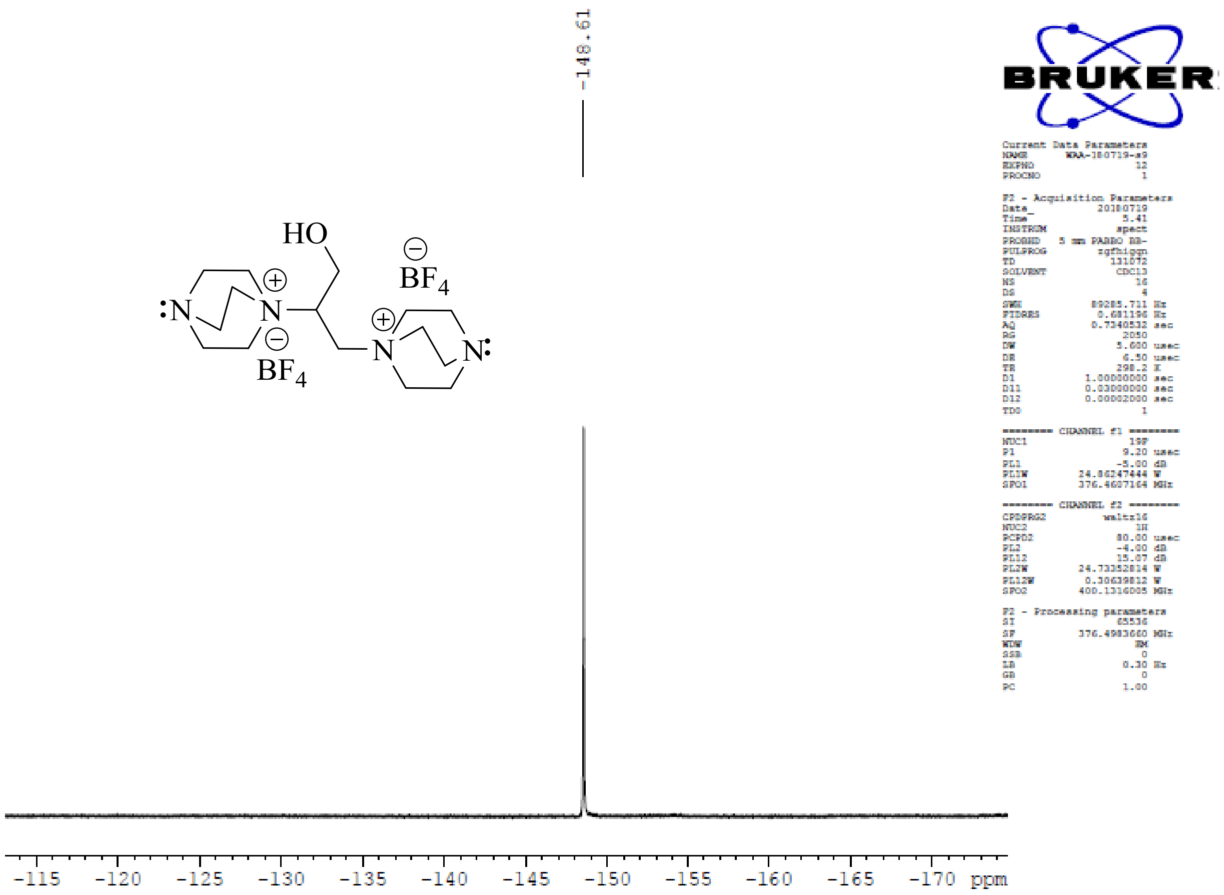

^{19}F NMR ($CDCl_3$): **3b**

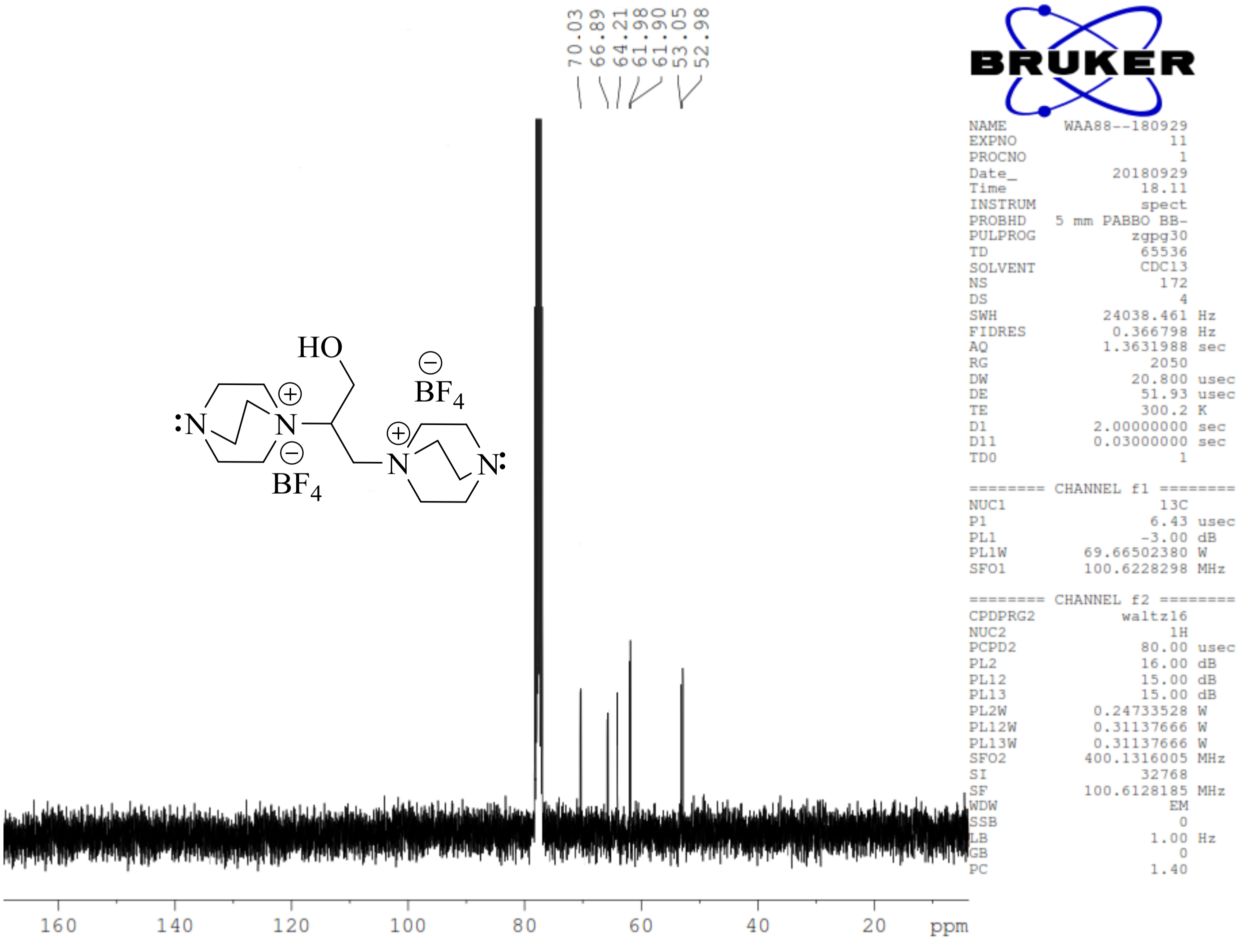

^{13}C NMR ($CDCl_3$): **3b**

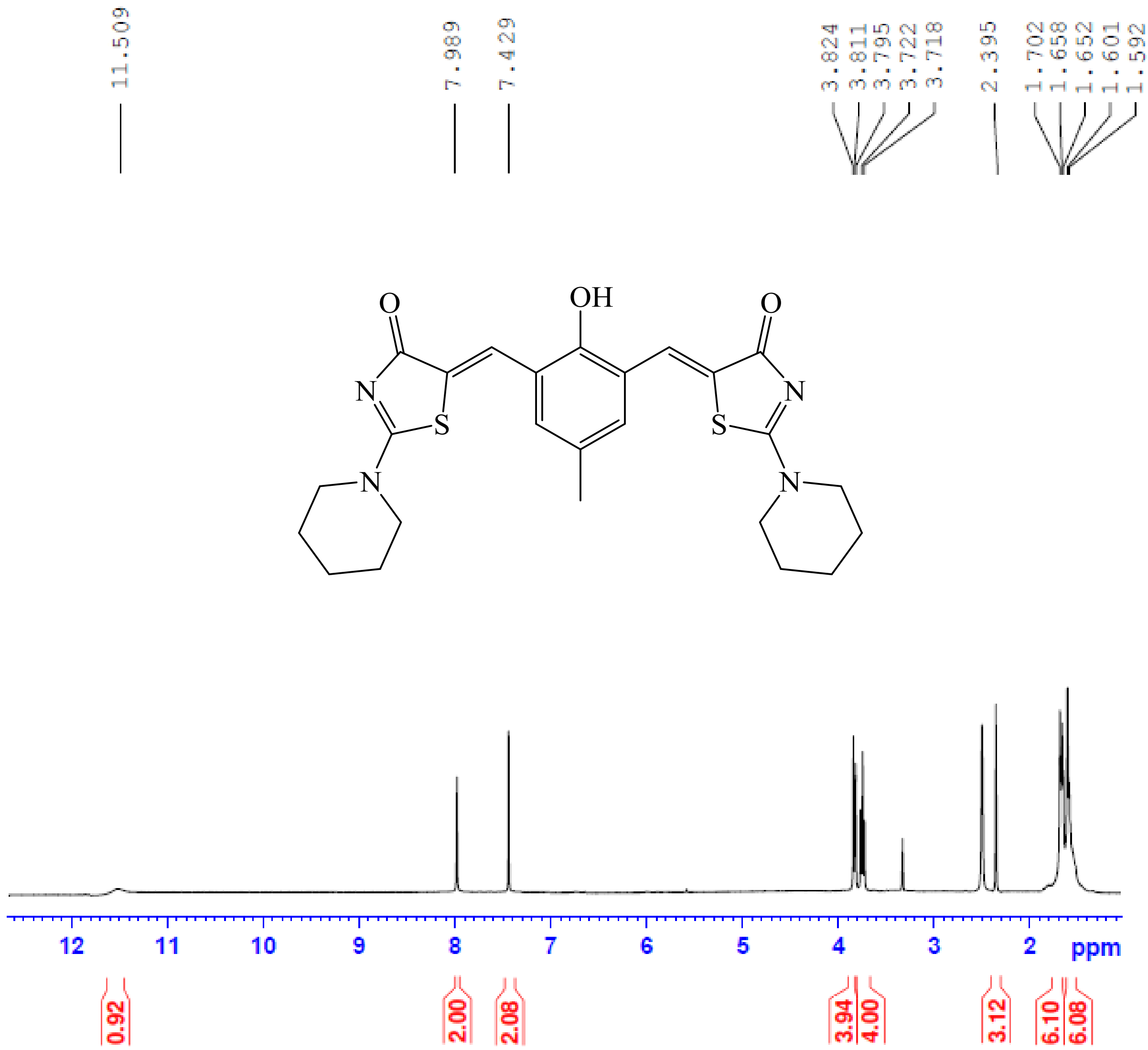

^1H NMR (DMSO-d_6): **7a**

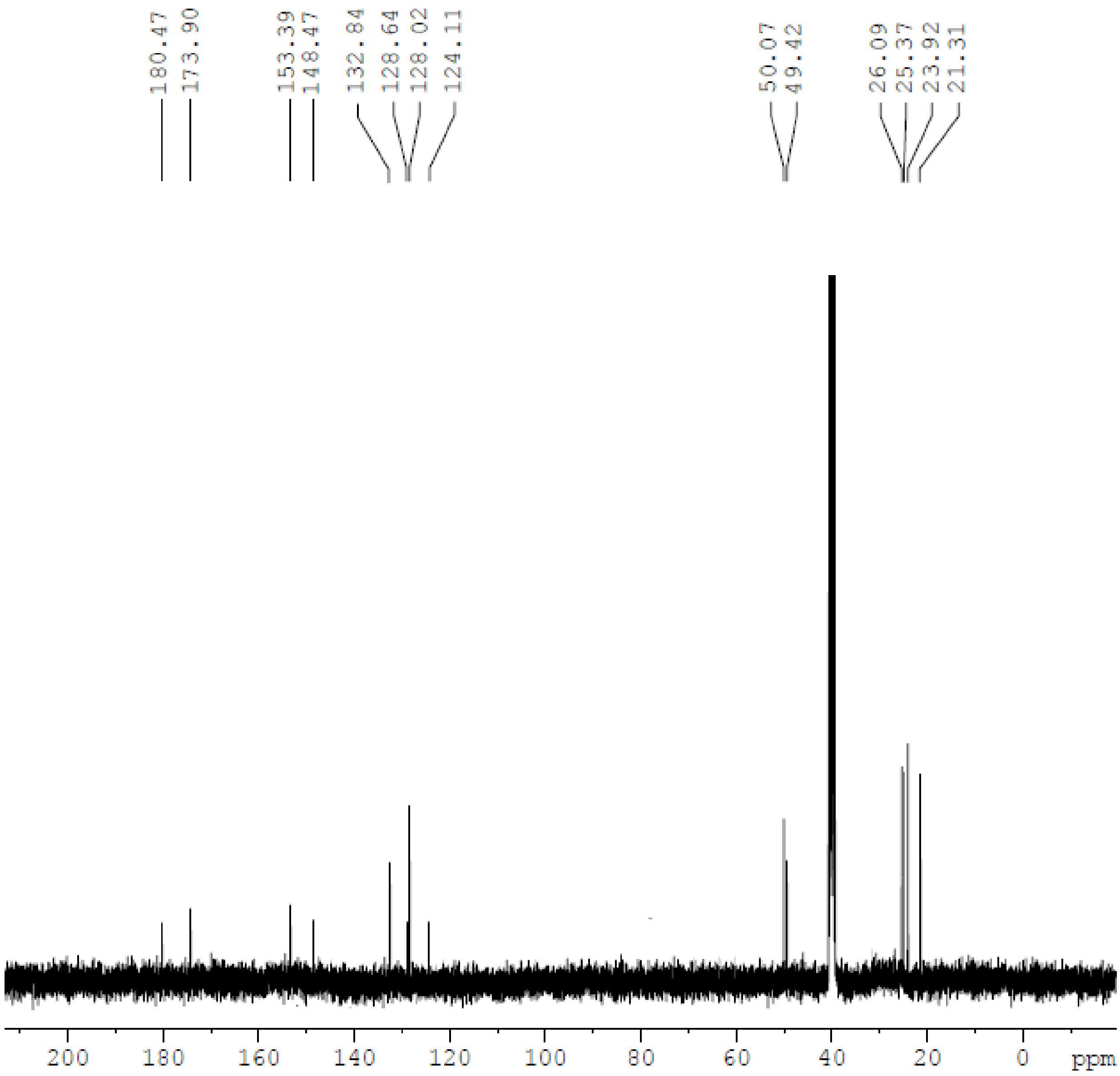

^{13}C NMR (DMSO-d_6): **7a**

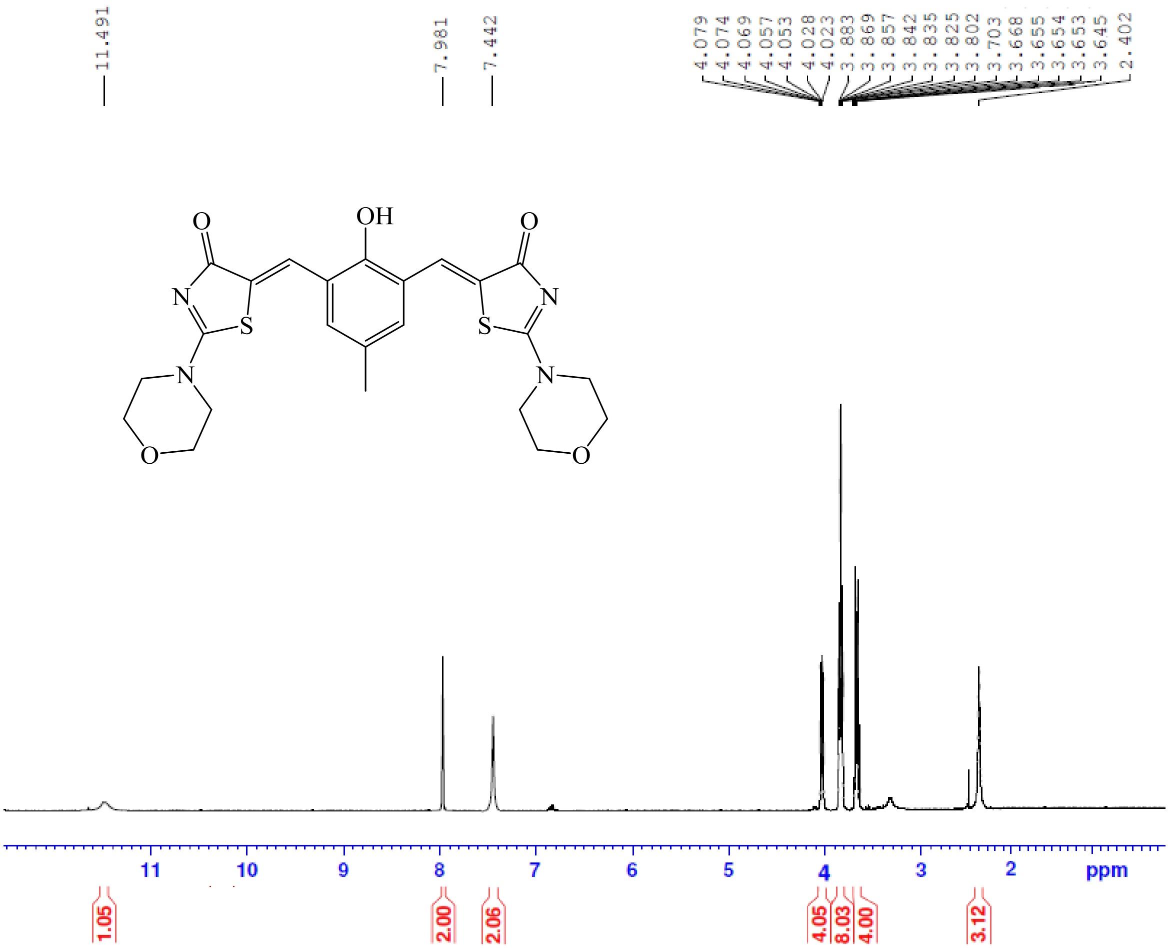

^{1}H NMR (DMSO-d_6): **7b**

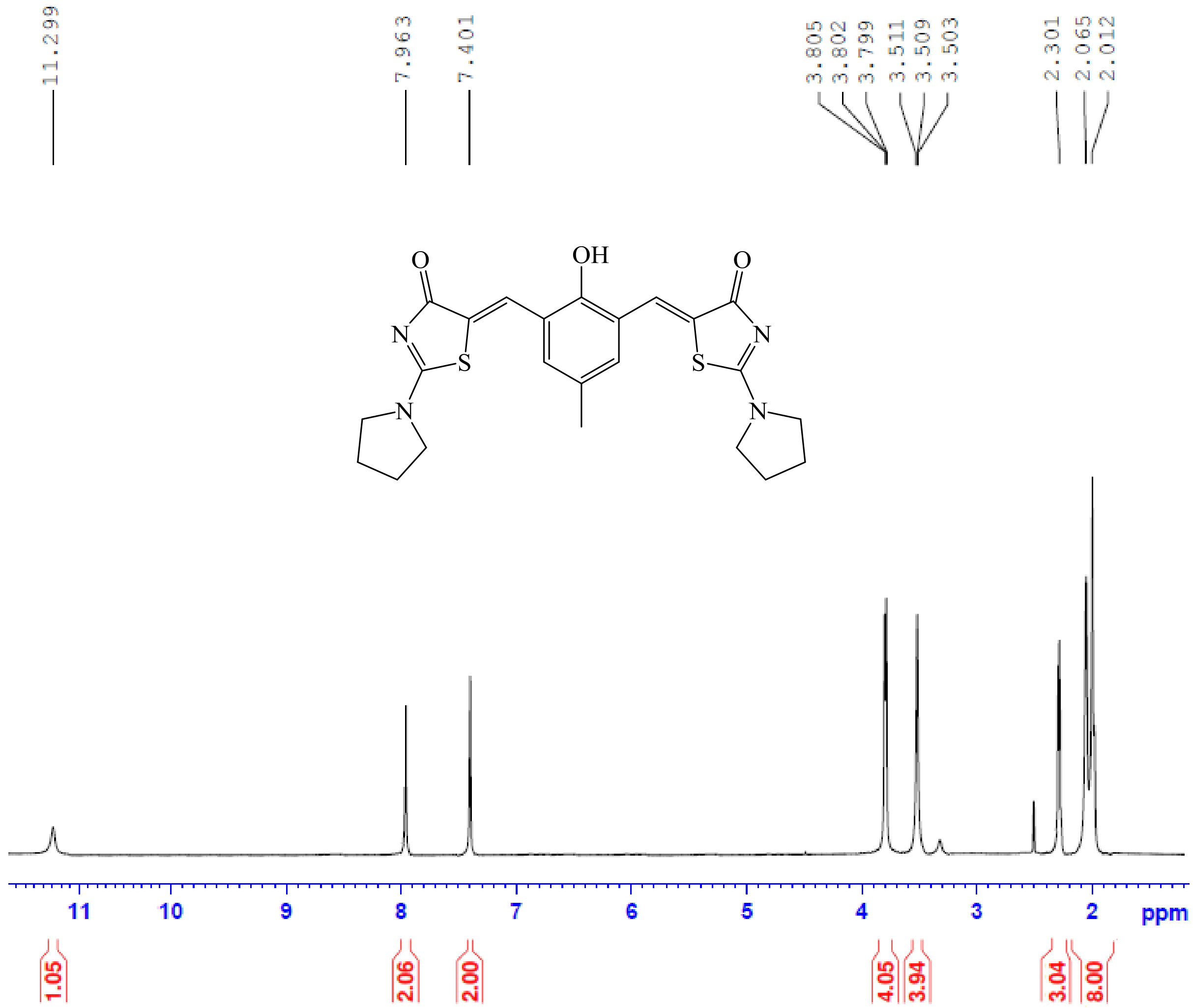

^{1}H NMR (DMSO-d_6): **7c**

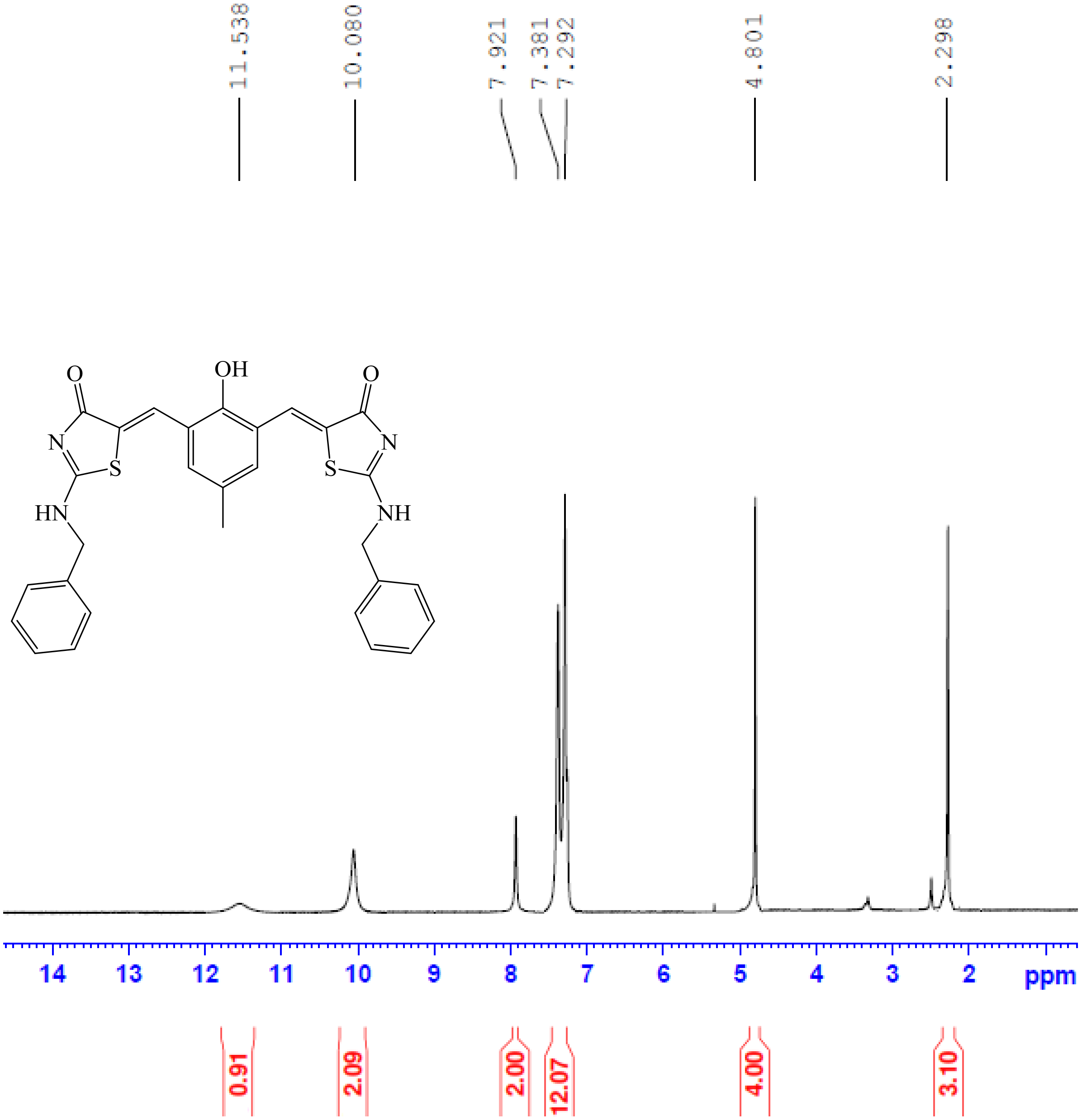

^{1}H NMR (DMSO-d_6): **7d**

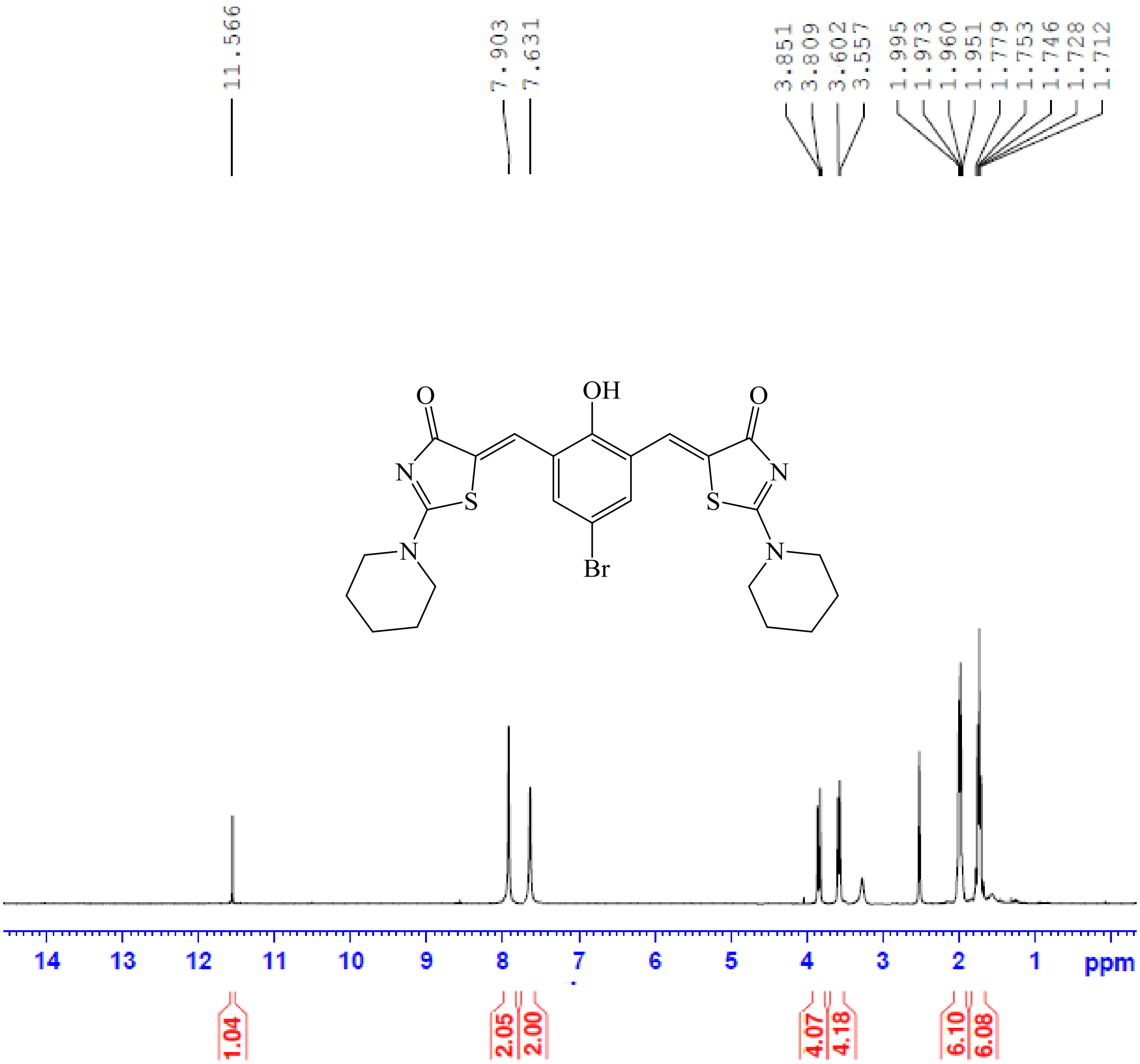

^{1}H NMR (DMSO-d_6): **7e**

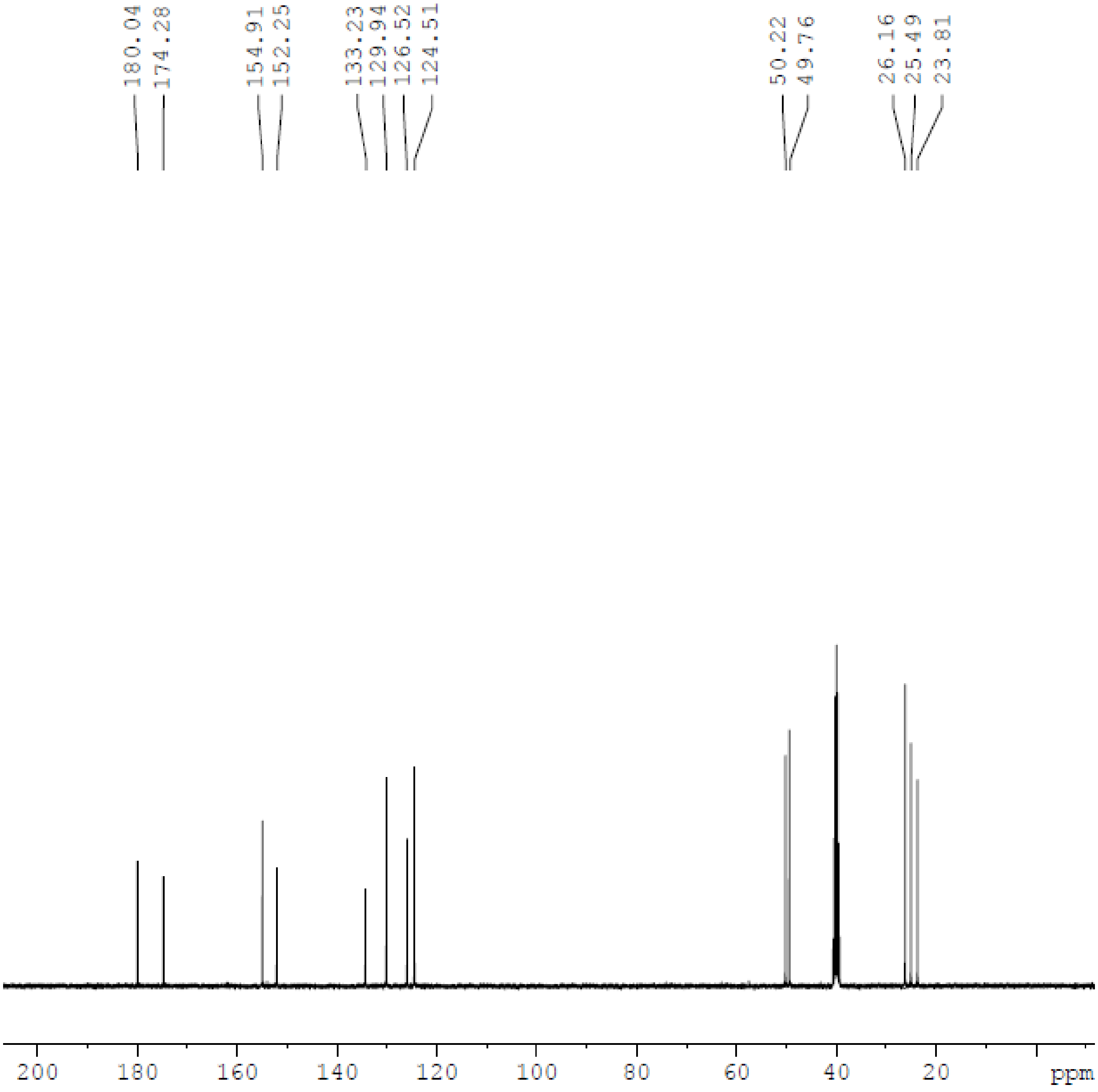

^{13}C NMR (DMSO-d_6): **7e**

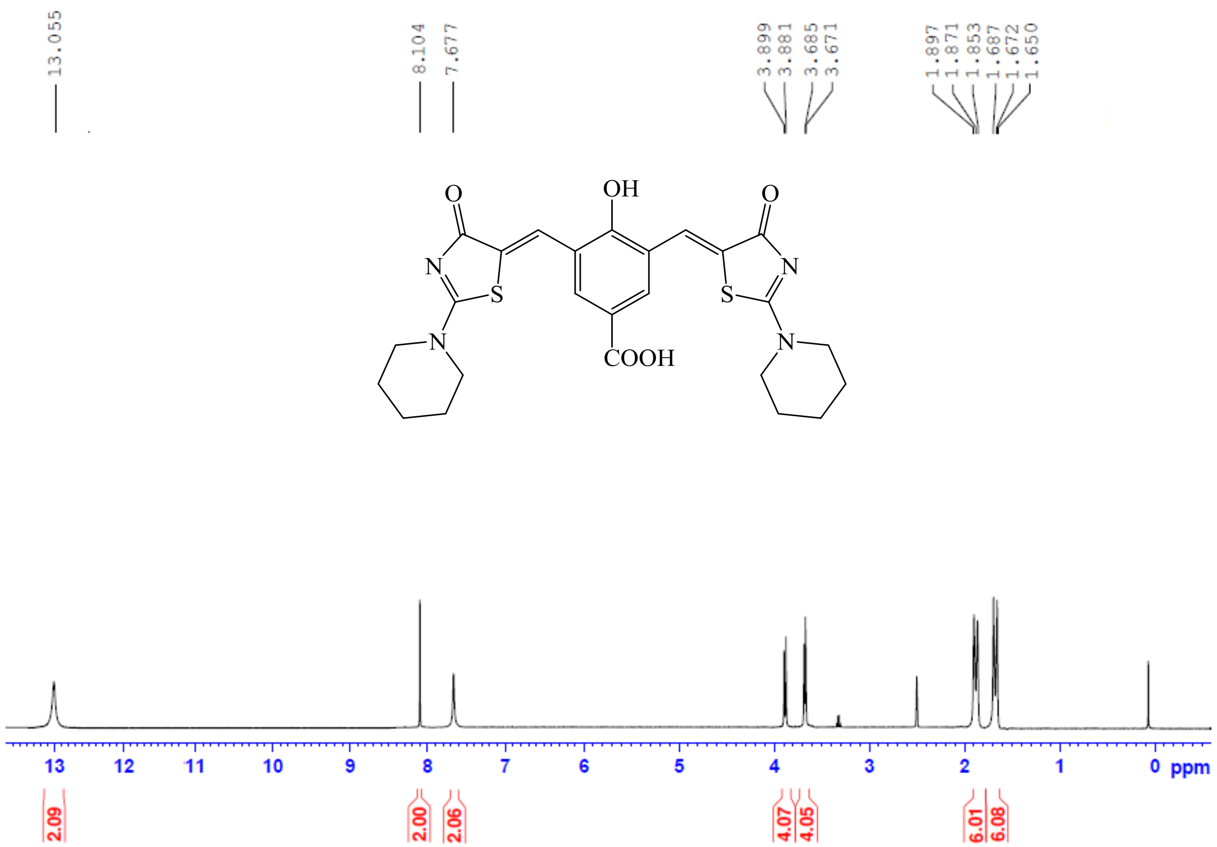

^{1}H NMR (DMSO-d_6): **7f**

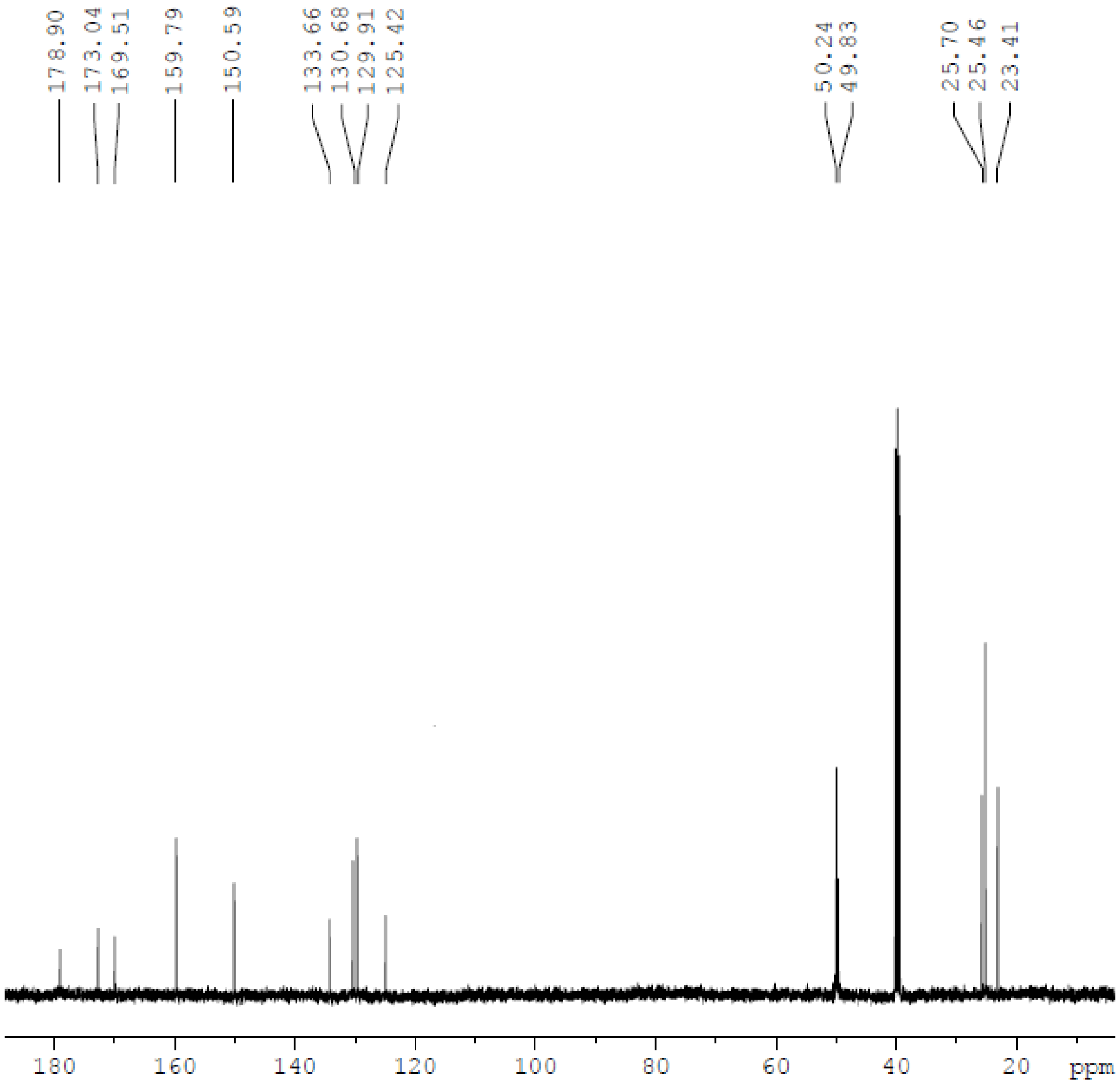

^{13}C NMR (DMSO-d_6): **7f**

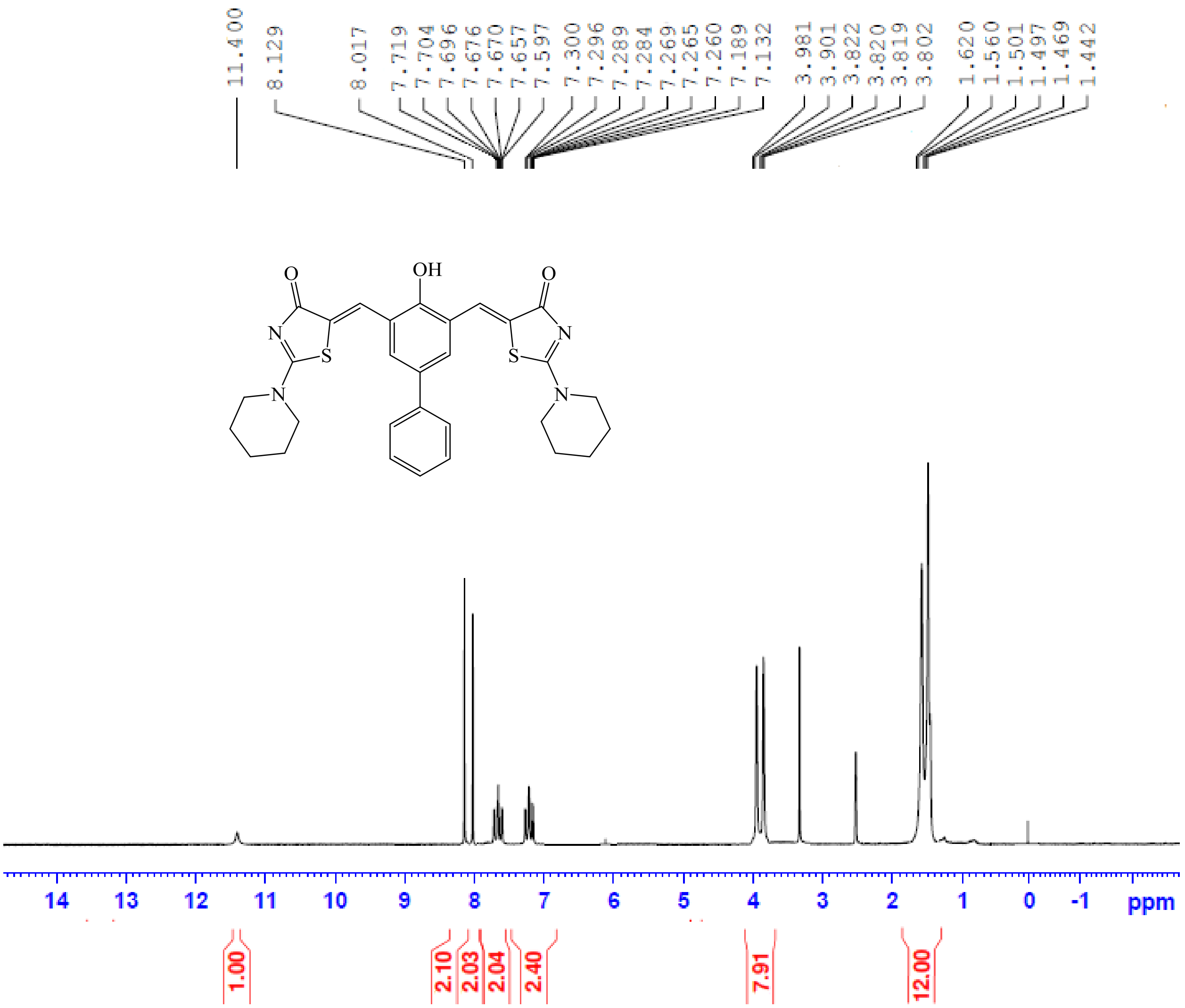

^{1}H NMR (DMSO-d_6): **7g**

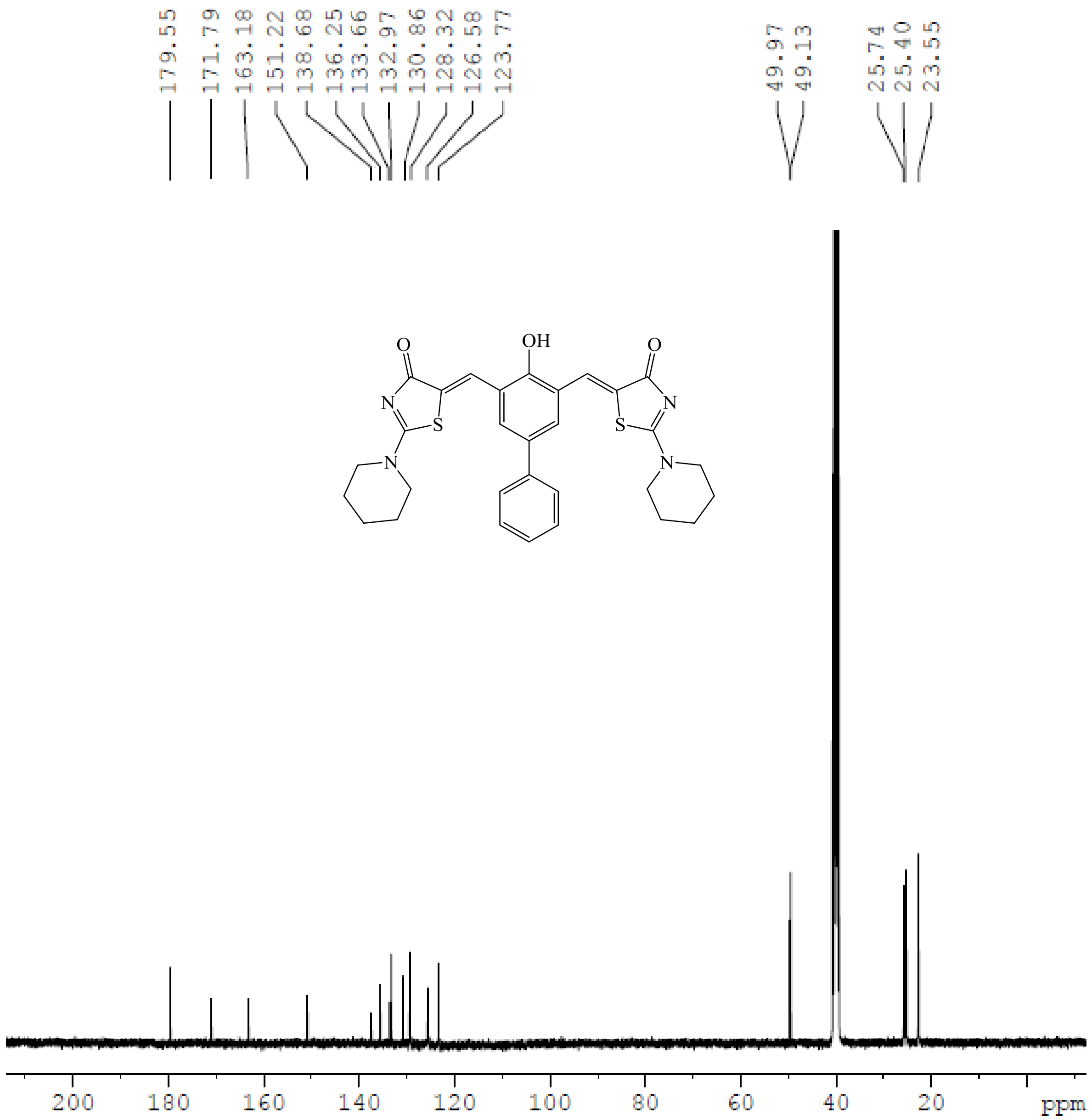

^{13}C NMR (DMSO-d_6): **7g**

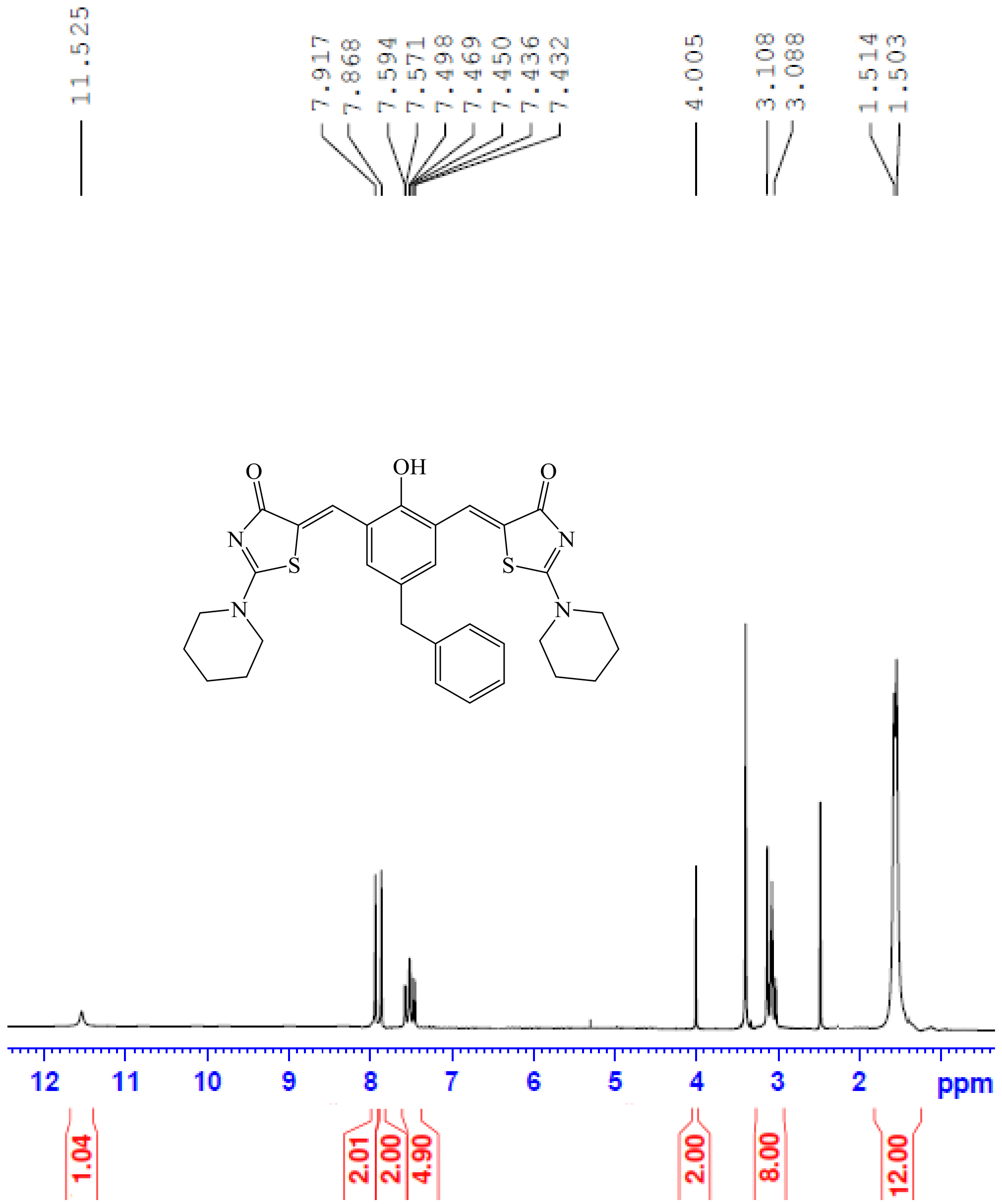

^{1}H NMR (DMSO-d_6): **7h**

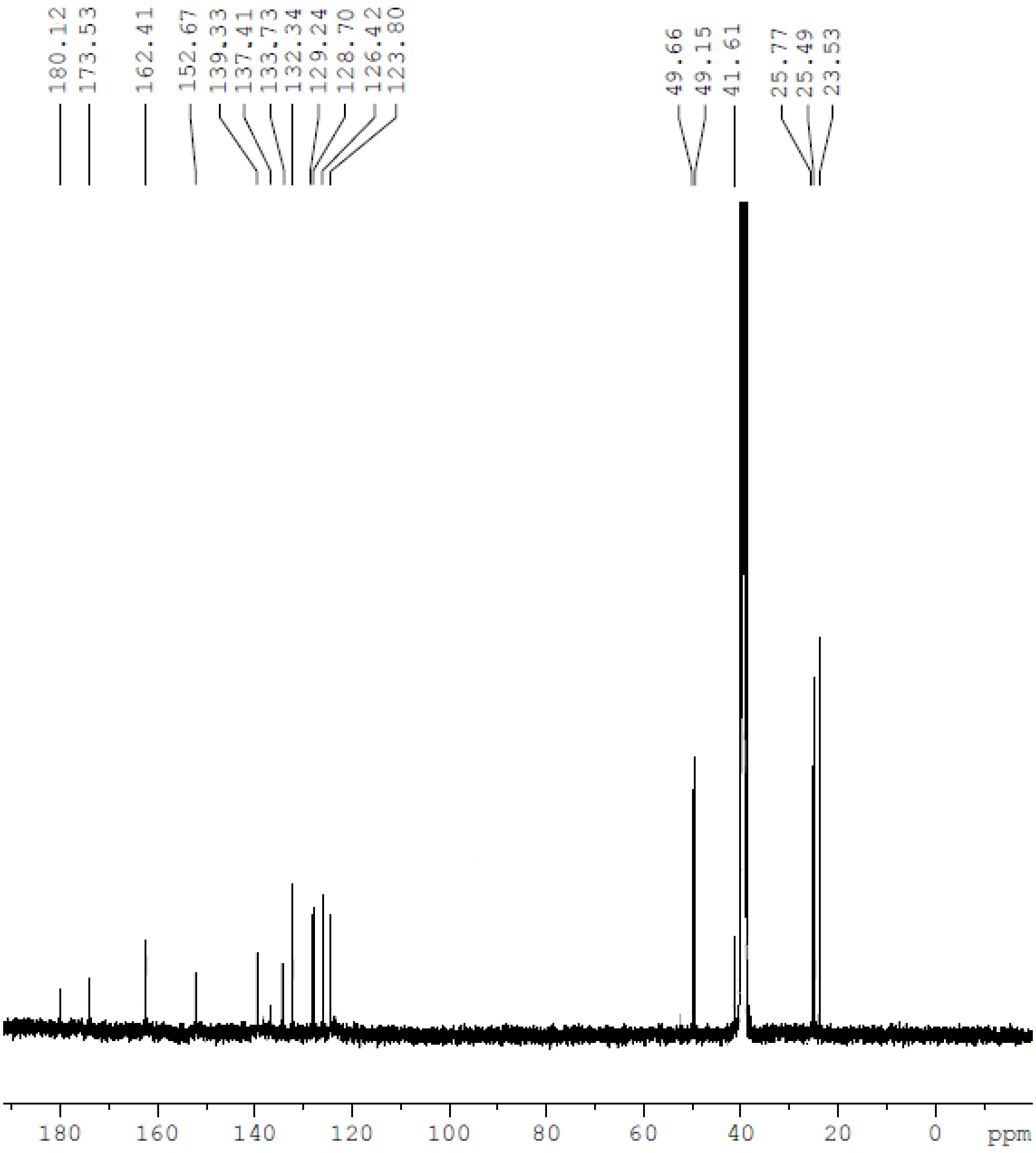

^{13}C NMR (DMSO-d_6): **7h**

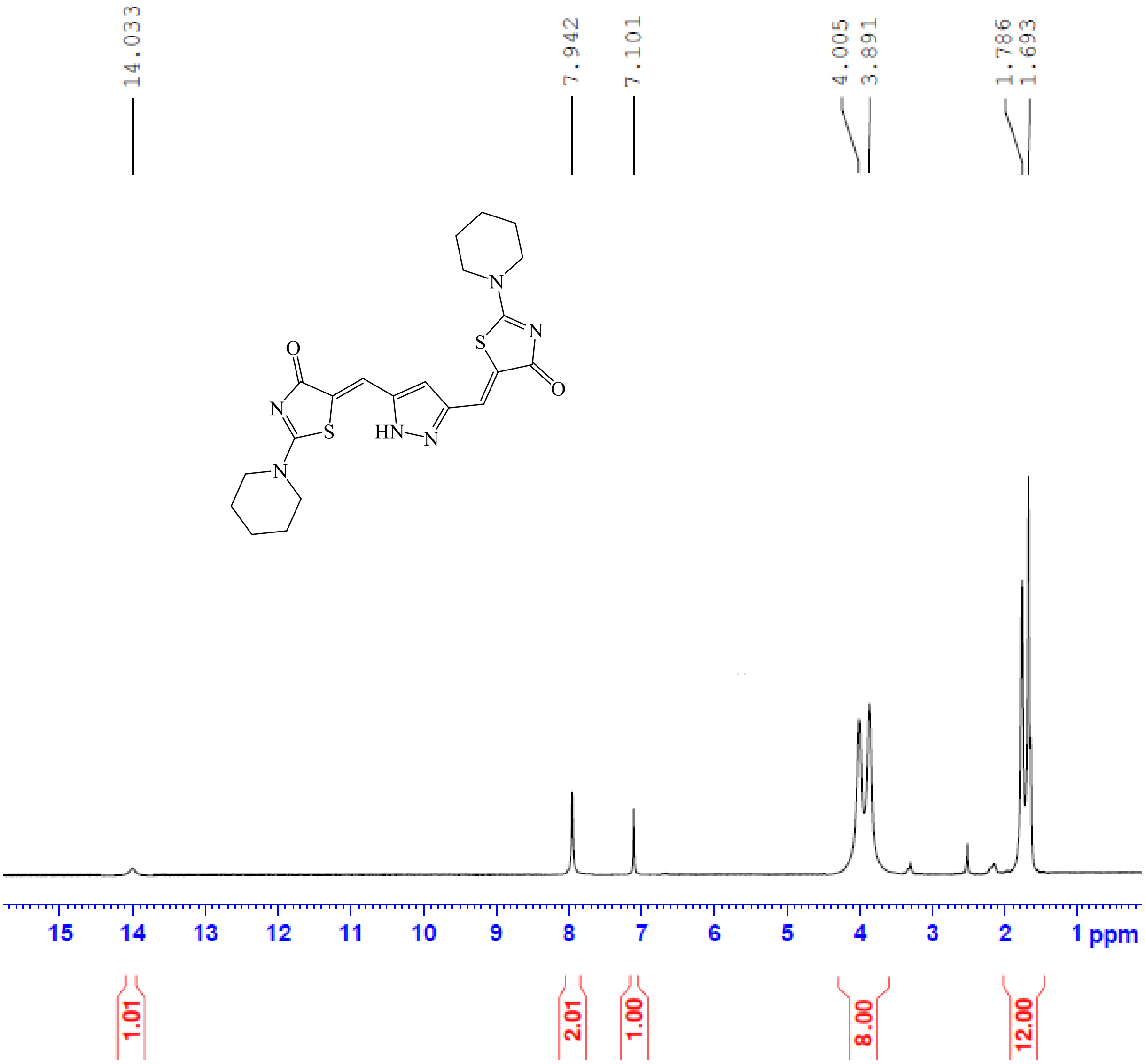

^{1}H NMR (DMSO-d_6): **7i**

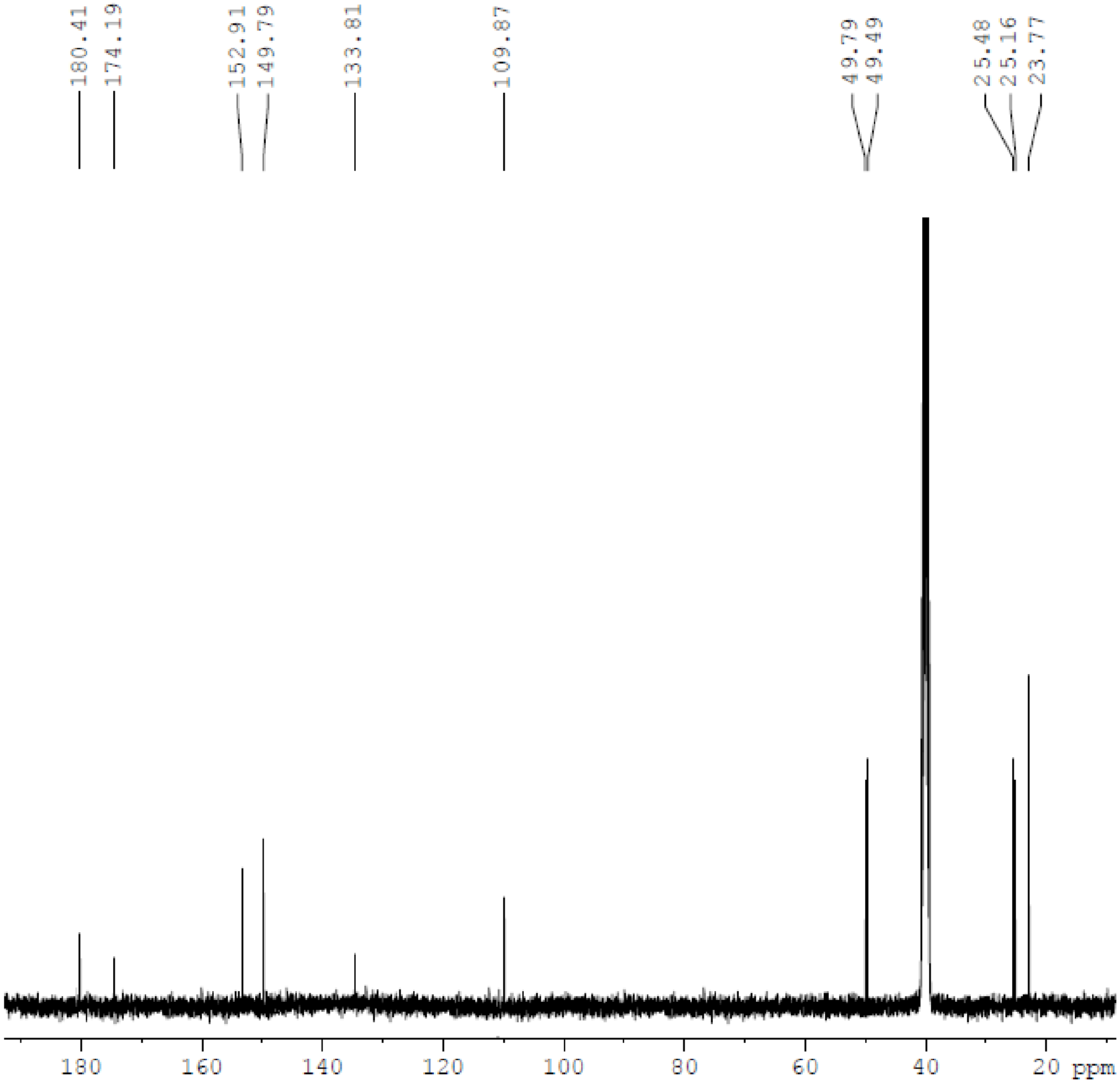

^{13}C NMR (DMSO-d_6): **7i**

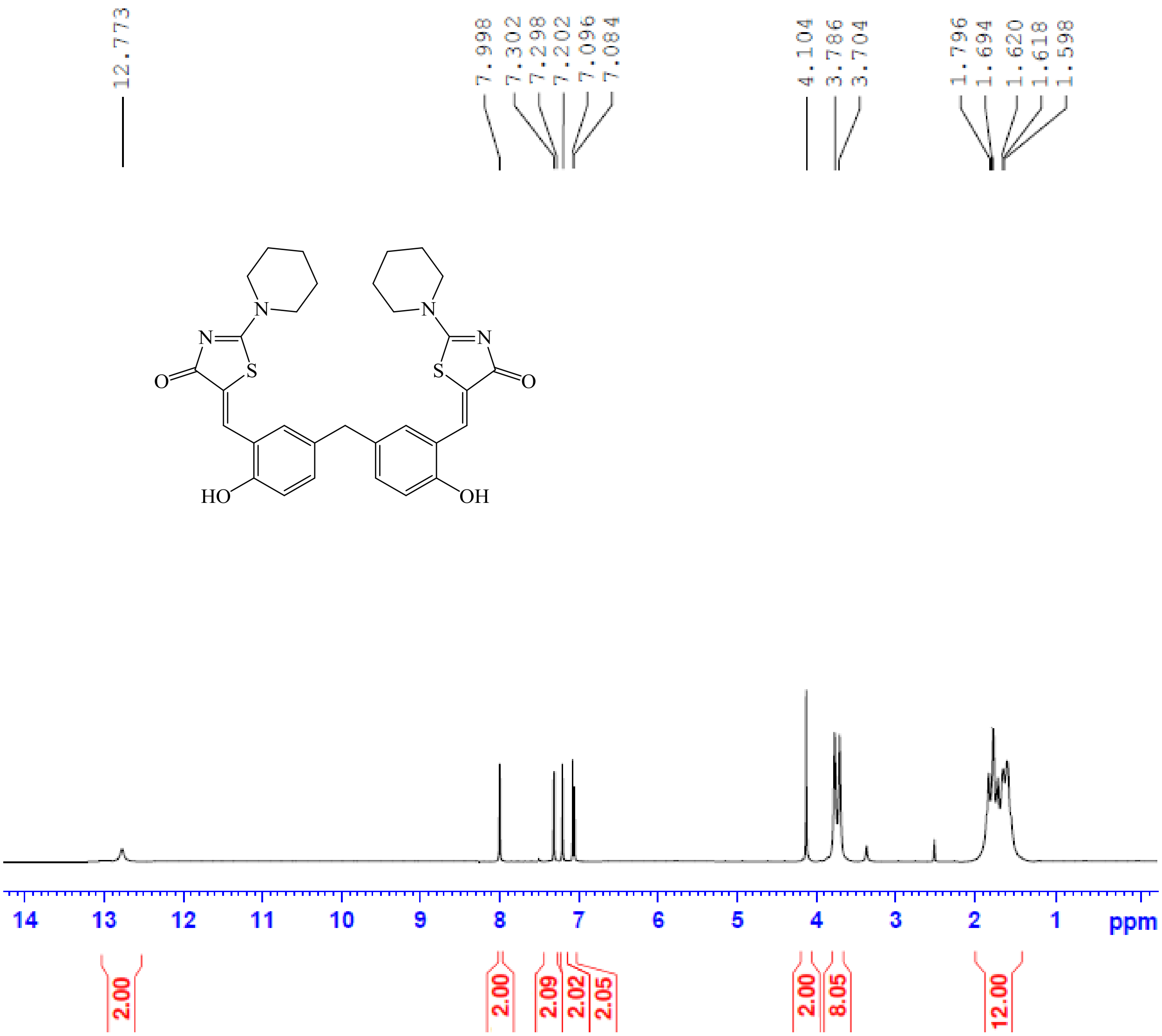

^{1}H NMR (DMSO-d_6): **7j**

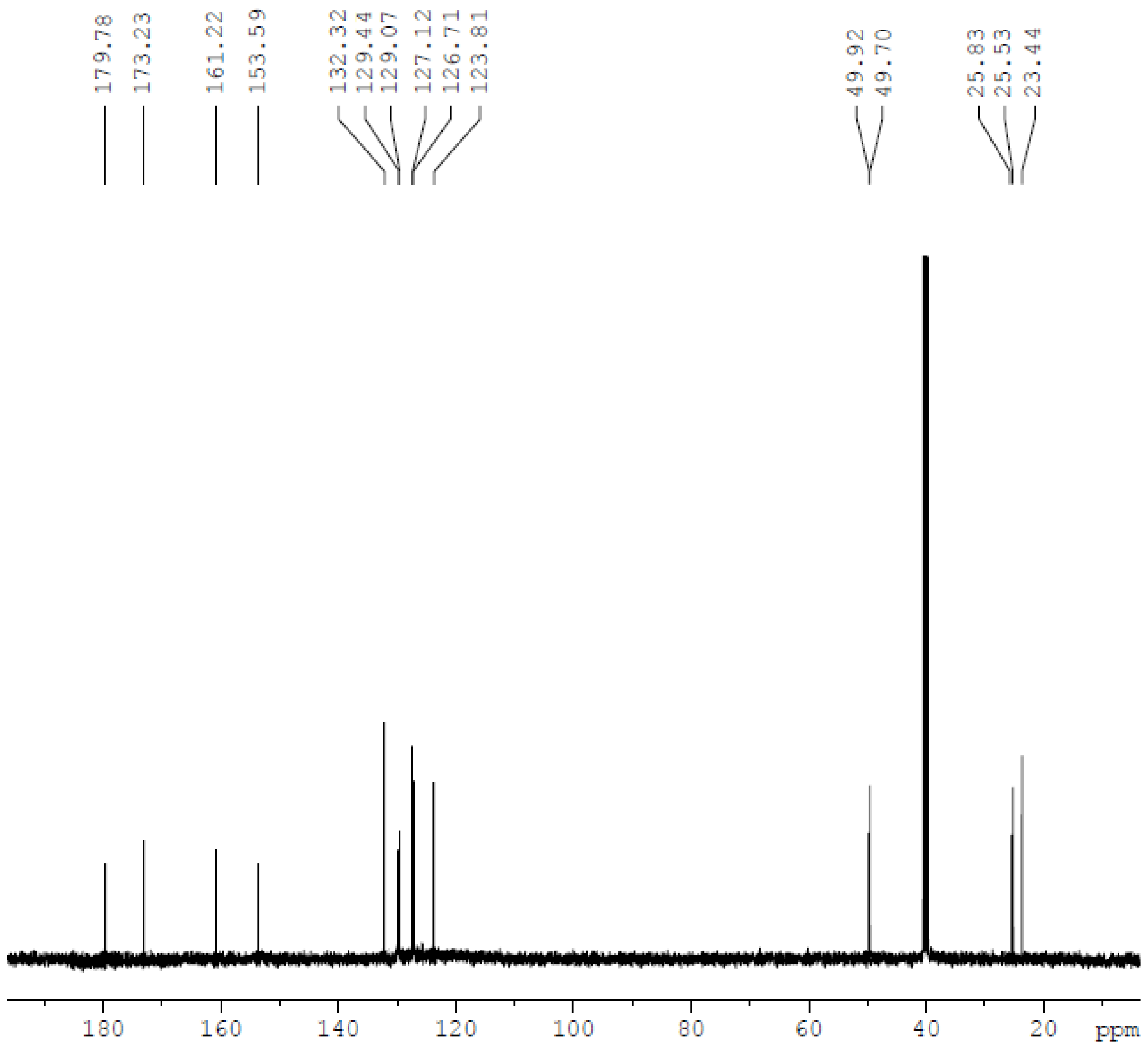

^{13}C NMR (DMSO-d_6): **7j**

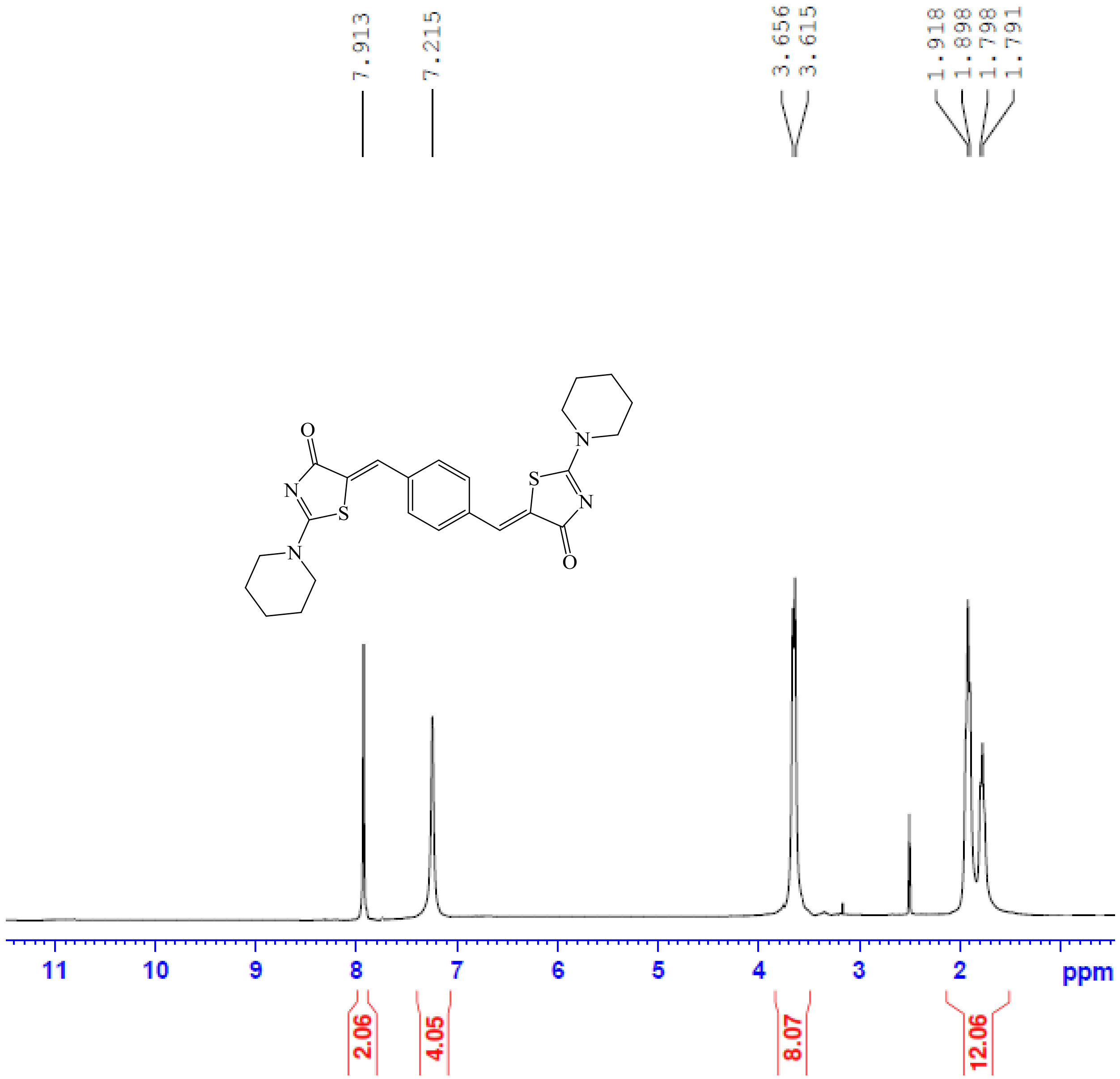

^{1}H NMR (DMSO-d_6): **7k**

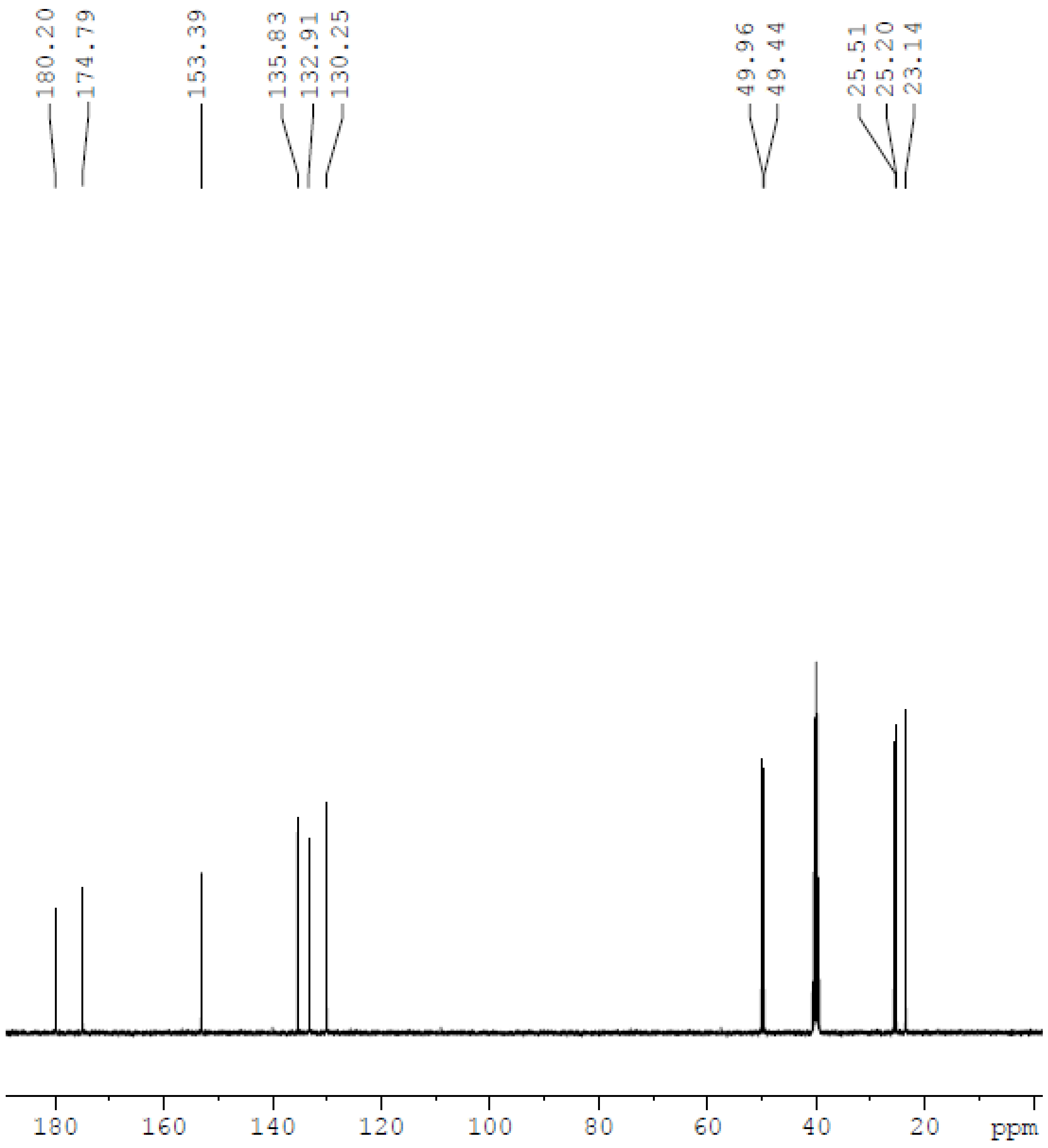

^{13}C NMR (DMSO-d_6): **7k**

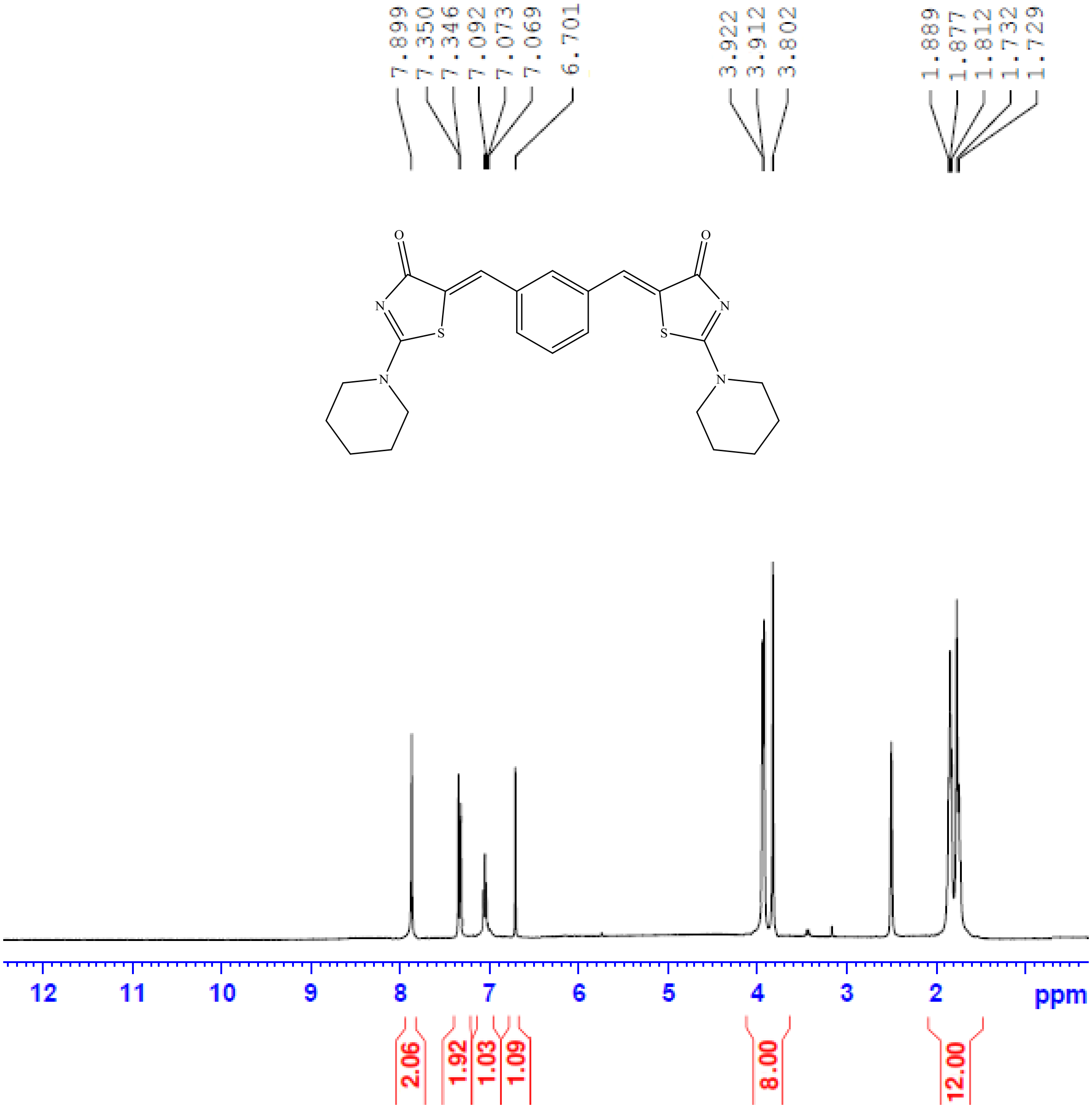

^{1}H NMR (DMSO-d_6): **7l**

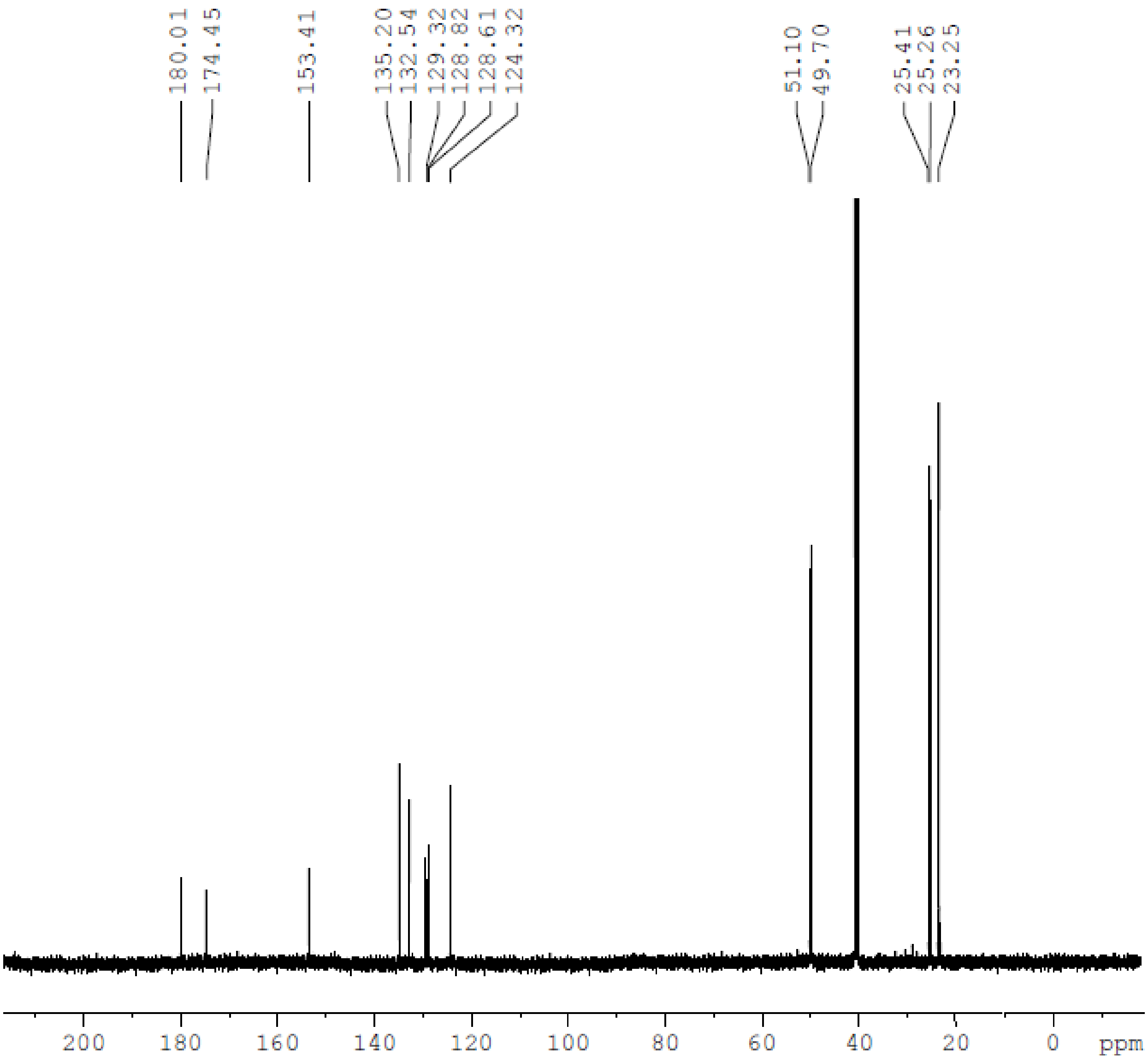

^{13}C NMR (DMSO-d_6): **7l**

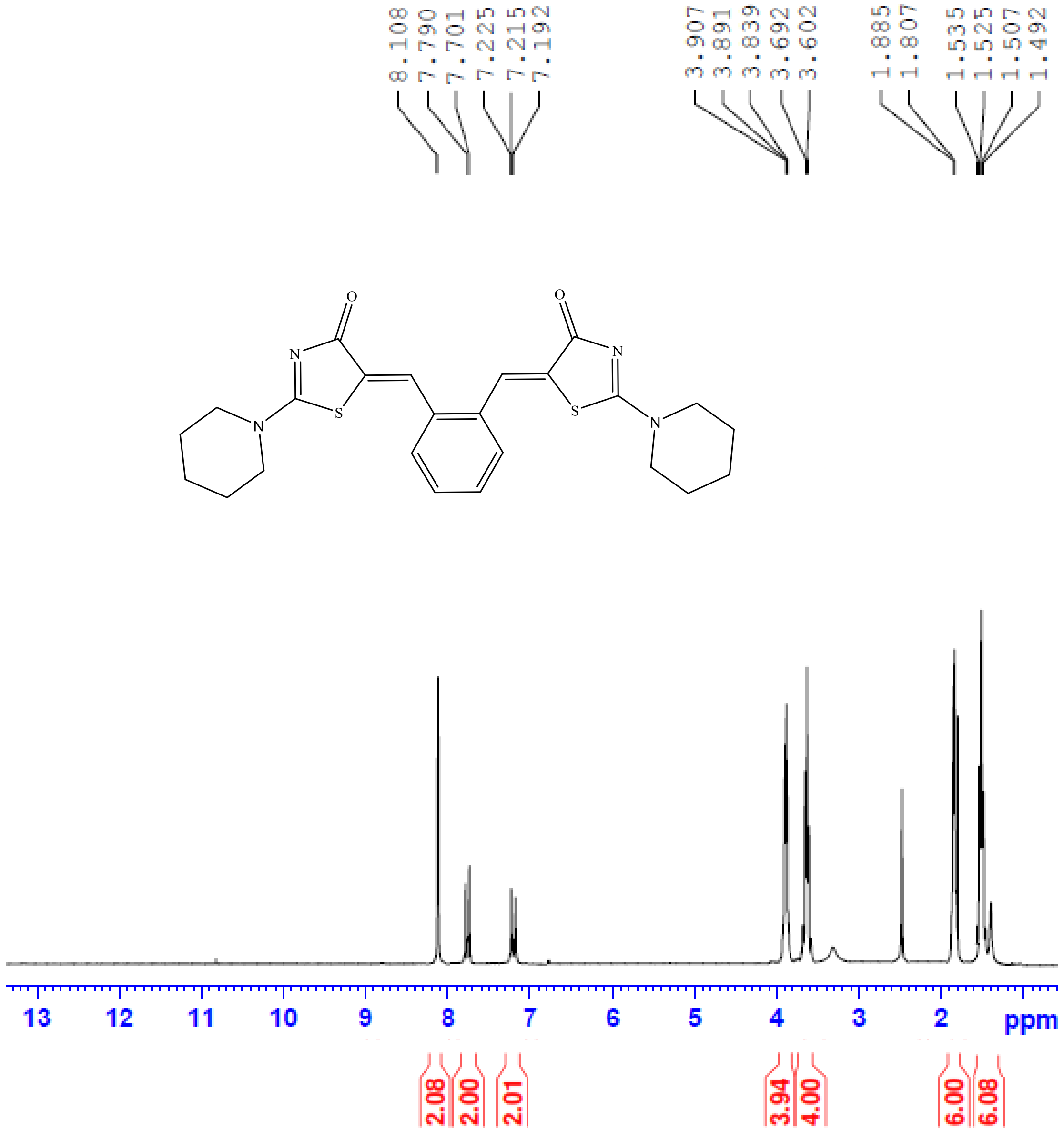

^{1}H NMR (DMSO-d_6): **7m**

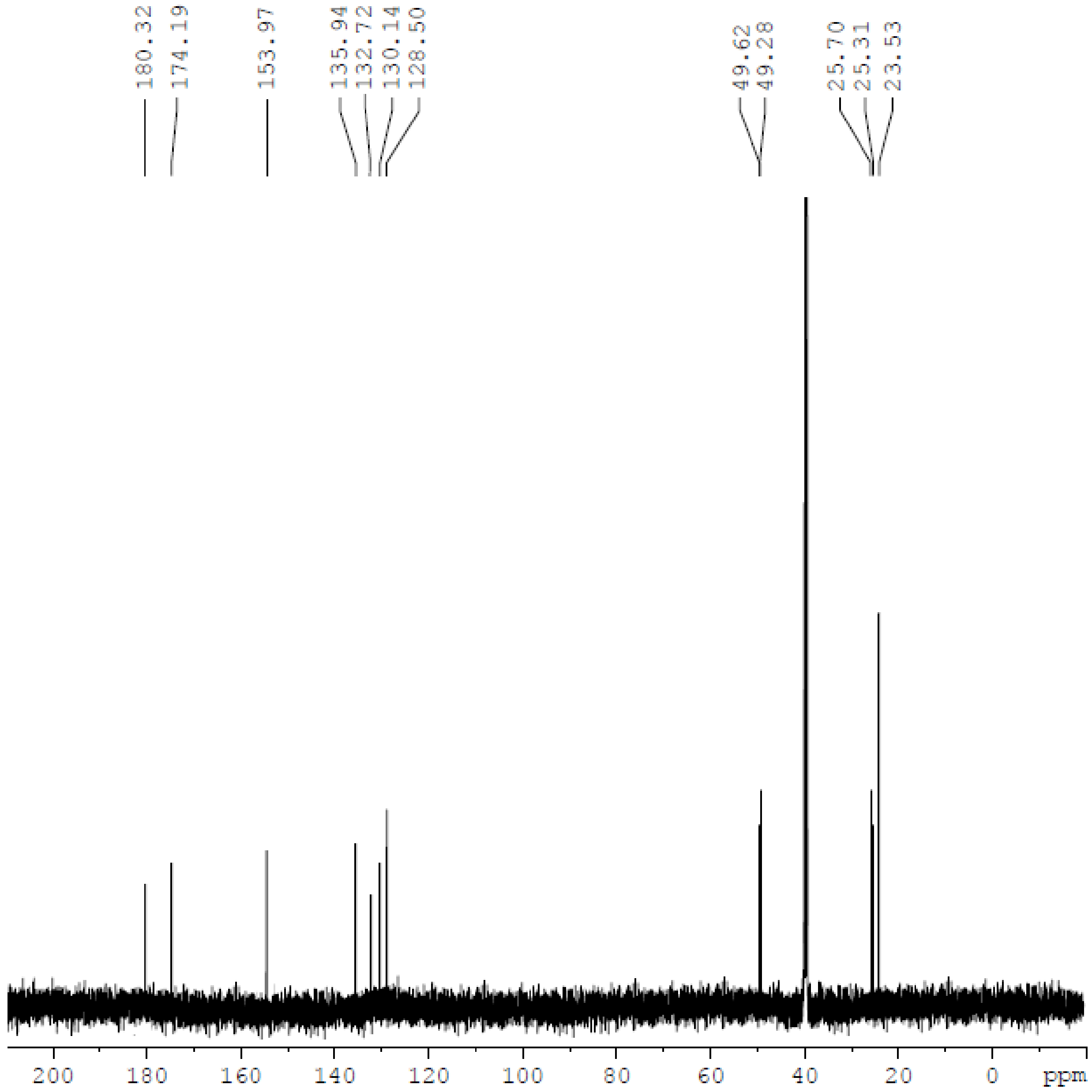

^{13}C NMR (DMSO-d_6): **7m**

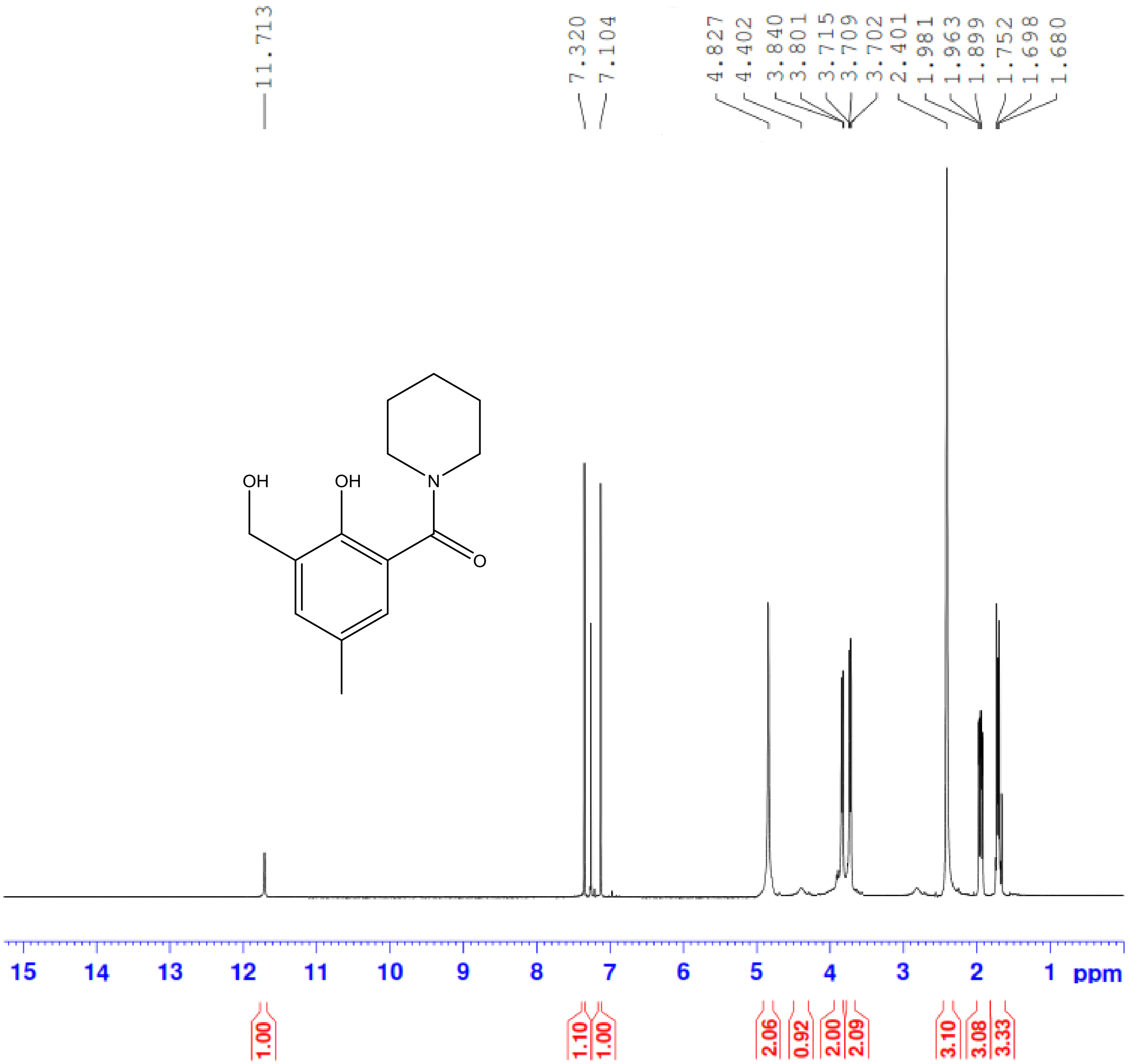
11.713
7.320
7.104
4.827
4.402
3.840
3.801
3.715
3.709
3.702
2.401
1.981
1.963
1.899
1.752
1.698
1.680
OH
OH
N
O
15 14 13 12 11 10 9 8 7 6 5 4 3 2 1 ppm
1.00
1.10
1.00
2.06
0.92
2.00
2.09
3.10
3.08
3.33

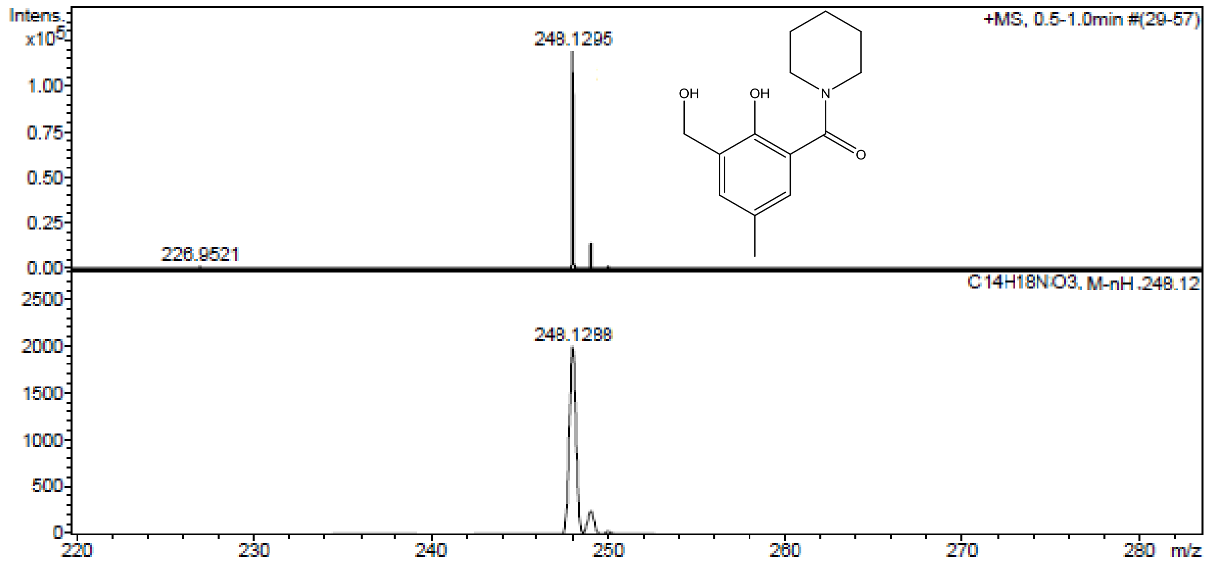

HRMS of (2-hydroxy-3-(hydroxymethyl)-5-methylphenyl)(piperidin-1-yl)methanone

Synthesis of 9a-m derivatives

(*Z*)-5-Benzylidene-2-(piperidin-1-yl)thiazol-4(5*H*)-one 9a

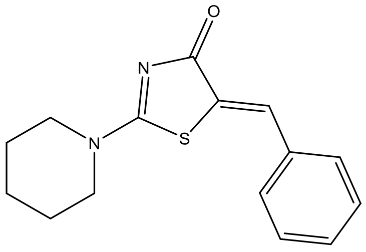

Yield 99%; yellow solid; mp = 215-216 °C; ^{1}H NMR (400 MHz, $CDCl_3$): δ 7.80 (s, 1H, =C*H*), 7.55 (d, 2H, J = 7.5 Hz, Ar-H), 7.48-7.44 (m, 2H, Ar-H), 7.39-7.35 (m, 1H, Ar-H), 4.05-4.00 (m, 2H, Piperidine-H), 3.58-3.61 (m, 2H, Piperidine-H), 1.69-1.81 (m, 6H, Piperidine-H); IR (KBr, cm^{-1}): ν_{max} 1705 (C=O), 1612 (C=N), 1599 (C=C); HRMS: *m*/*z* [M + H]$^+$ calcd: 273.1062, found 273.1054.

(*Z*)-5-(4-Chlorobenzylidene)-2-(piperidin-1-yl)thiazol-4(5*H*)-one 9b

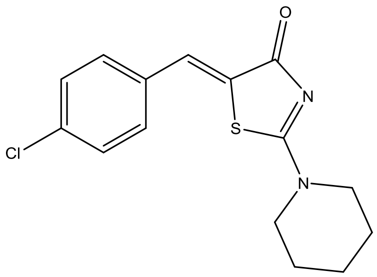

Yield 99%; white solid, mp = 206-208 °C; ^{1}H NMR (400 MHz, DMSO-*d6*) δ: 7.99 (s, 1H, =C*H*), 7.53 (brs, 4H, Ar-H), 3.89 (brs, 4H, Piperidine-H), 1.94-1.89 (m, 6H, Piperidine-H); IR (KBr, cm^{-1}): ν_{max} 1668 (C=O), 1611 (C=N), 1587 (C=C); HRMS: *m*/*z* [M]$^+$ calcd: 306.0592, found 306.0598.

(*Z*)-5-(4-Methylbenzylidene)-2-(piperidin-1-yl)thiazol-4(5*H*)-one 9c

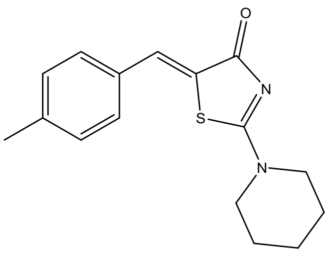

Yield 99%; yellowish solid; mp = 154-156 °C; ^{1}H NMR (400 MHz, $CDCl_3$): δ 7.56 (s, 1H, =C*H*), 7.48 (d, J = 8.1 Hz, 2H, Ar-H), 7.27 (d, J = 7.8 Hz, 2H, Ar-H), 3.88-3.84 (m, 2H, Piperidine-H), 3.59 (br, 2H, Piperidine-H), 2.31 (s, 3H, CH_3), 1.65 ppm (br, 6H, Piperidine-H); IR (KBr, cm^{-1}): ν_{max} 1689 (C=O), 1621 (C=N), 1603 (C=C); HRMS: *m*/*z* $[M]^+$ calcd: 286.1141, found 286.1147.

(*Z*)-5-(4-Methoxybenzylidene)-2-(piperidin-1-yl)thiazol-4(5*H*)-one 9d

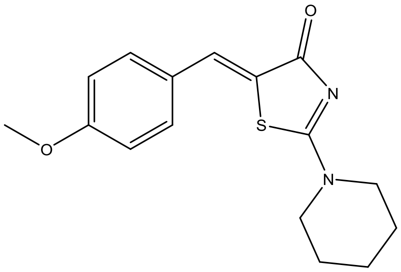

Yield 99%; white solid; mp = 200-201 ^{o}C; ^{1}H NMR (400 MHz; $CDCl_3$): δ 7.71 (s, 1H, =C*H*), 7.40 (d, J = 8.8 Hz, 2H, Ar-H), 6.86 (d, J = 9 Hz, 2H, Ar-H), 3.96-3.92 (m, 2H, Piperidine-H), 3.65 (s, 3H, OCH_3), 3.50 (m, 2H, Piperidine-H), 1.72 (m, 6H, Piperidine-H); IR (KBr, cm^{-1}): ν_{max} 1683 (C=O), 1608 (C=N), 1598 (C=C); HRMS: *m*/*z* $[M + H]^+$ calcd: 303.1169, found 303.1184.

(*Z*)-5-(2-Bromobenzylidene)-2-(piperidin-1-yl)thiazol-4(5*H*)-one 9e

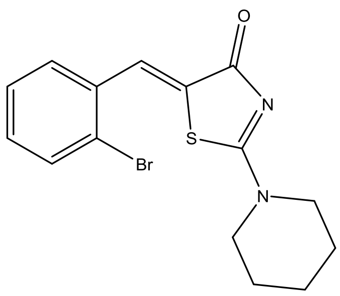

Yield 97%; yellow solid, mp = 165-168°C; ^{1}H NMR (400 MHz, $CDCl_3$) δ: 8.02 (s, 1H, =C*H*), 7.66 (d, J = 7.7 Hz, 1H, Ar-H), 7.56 (d, J = 7.7 Hz, 1H, Ar-H), 7.38 (t, J = 7.4 Hz, 1H, Ar-H), 7.22 (t, J = 7.7 Hz, 1H, Ar-H), 4.01 (s, 2H, Piperidine-H), 3.57 (s, 2H, Piperidine-H), 1.76 (br, 6H, Piperidine-H); IR (KBr, cm^{-1}): ν_{max} 1698 (C=O), 1611 (C=N), 1599 (C=C); HRMS: *m*/*z* $[M]^+$ calcd: 350.0089, found 350.0084.

(*Z*)-4-((4-Oxo-2-(piperidin-1-yl)thiazol-5(4*H*)-ylidene)methyl)benzonitrile 9f

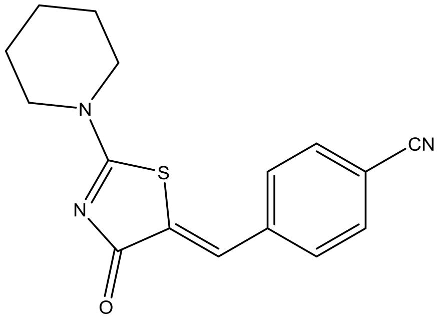

Yield 98%; yellow solid; mp = 193-195; ^{1}H NMR (400 MHz, DMSO-d_6): δ 7.97 (d, J = 8.7 Hz, 2H, Ar-H), 7.80 (d, J = 8.7 Hz, 2H, Ar-H), 7.67 (s, 1H, =C*H*), 3.96–3.90 (m, 2H, Piperidine-H), 3.65-366 (m, 2H, br s, Piperidine-H), 1.66-167 (m, 6H, Piperidine-H); IR (KBr, cm^{-1}): ν_{max} 2223 (C≡N), 1689 (C=O), 1617 (C=N), 1610 (C=C); HRMS: *m*/*z* [M + H]$^+$ calcd: 298.1015, found 298.1019.

(*Z*)-5-(Benzo[*d*][1,3]dioxol-4-ylmethylene)-2-(piperidin-1-yl)thiazol-4(5*H*)-one 9g

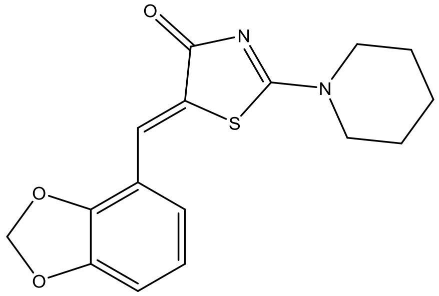

Yield 98%; white solid; mp = 185-186 ^{o}C; ^{1}H NMR (400 MHz; CDCl$_3$): δ 7.69 (s, 1H, =C*H*), 7.01 (d, J = 8.8 Hz, 1H, Ar-H), 6.97 (m, 1H, Ar-H); 6.80 (d, J = 8.8 Hz, 1H, Ar-H); 6.03 (s, 2H, C*H*$_2$), 3.99-3.96 (m, 2H, Piperidine-H), 3.49 (m, 2H, Piperidine-H), 1.79-1.73 (m, 6H, Piperidine-H); IR (KBr, cm^{-1}): ν_{max} 1699 (C=O), 1605 (C=N), 1585 (C=C); HRMS: *m*/*z* [M + H]$^+$ calcd: 317.0960, found 317.0957.

(*Z*)-5-(3,4-Dimethoxybenzylidene)-2-(piperidin-1-yl)thiazol-4(5*H*)-one 9h

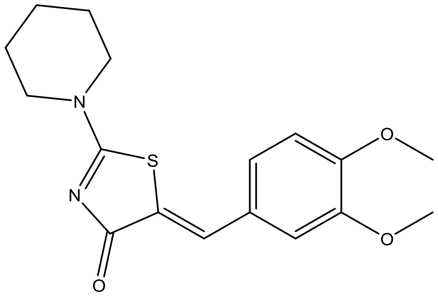

Yield 97%; yellowish solid; mp = 198-199°C; ^{1}H NMR (400 MHz, CDCl$_3$) δ: 7.72 (s, 1H, =C*H*), 7.13-7.14 (dd, J = 8.7, 1.7 Hz, 1H, Ar-H), 7.01 (d, J = 1.7 Hz, 1H, Ar-H), 6.93 (d, J = 8.7 Hz, 1H, Ar-H),

3.99-4.01 (t, J = 5.9 Hz, 2H, Piperidine-H), 3.90 (d, 6H, -OCH_3), 3.56 (br, 2H, piperidine-H), 1.78 (br, 6H, piperidine-H); IR (KBr, cm^{-1}): ν_{max} 1655 (C=O), 1614 (C=N), 1601 (C=C); HRMS: *m*/*z* $[M + H]^+$ calcd: 333.1271, found 333.1274.

(*Z*)-5-(4-Aminobenzylidene)-2-(piperidin-1-yl)thiazol-4(5*H*)-one 9i

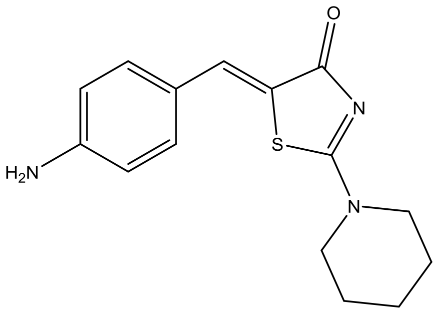

Yield 96%; yellow solid, mp = 213-215 °C; ^1H NMR (400 MHz, DMSO-*d6*) δ: 7.43 (s, 1H, =C*H*), 7.25 (d, J = 8.7 Hz, 2H, Ar-H), 6.69 (d, J = 8.8 Hz, 2H, Ar-H), 5.90 (br, 2H, NH_2), 3.92 (br, 2H, Piperidine-H), 3.55 (br, 2H, Piperidine-H), 1.60 (br, 6H, Piperidine-H); IR (KBr): 1688 (C=O), 1609 (C=N), 1597 (C=C); HRMS: *m*/*z* $[M + Na]^+$ calcd: 310.0991, found 310.0987.

(*Z*)-5-(4-Hydroxy-3-methoxybenzylidene)-2-(piperidin-1-yl)thiazol-4(5*H*)-one 9j

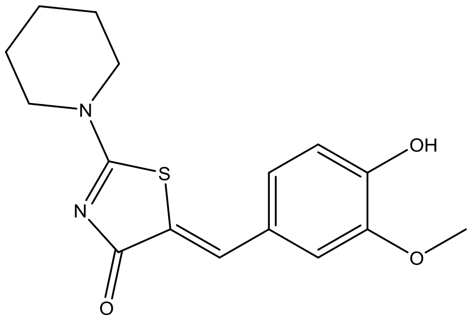

Yield 98%; yellow solid; mp = 193-195°C; ^1H NMR (400 MHz, $CDCl_3$) δ: 7.70 (s, 1H, =C*H*), 7.10-712 (dd, J = 8.5, 1.7 Hz, 1H, Ar-H), 7.03-7.00 (m, 2H, Ar-H), 4.00-4.02 (t, J = 5.9 Hz, 2H, Piperidine-H), 3.96 (s, 3H, OCH_3), 3.59 (br, 2H, Piperidine-H), 1.76 (br, 6H, Piperidine-H); IR (KBr, cm^{-1}): ν_{max} 1672 (C=O), 1598 (C=N, C=C); HRMS: *m*/*z* $[M]^+$ calcd: 318.1036, found 318.1038.

(*Z*)-2-(Piperidin-1-yl)-5-(pyridin-4-ylmethylene)thiazol-4(5*H*)-one 9k

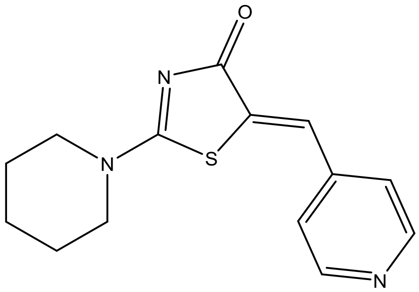

Yield 98%; yellow solid; mp = 171-173 °C; ^{1}H NMR (400 MHz, $CDCl_3$) δ: 8.67 (d, J = 5.0 Hz, 2H, Ar-H), 7.56-7.52 (m, 3H, Ar-H + =C*H*), 3.89 (br, 2H, Piperidine-H), 3.63 (br, 2H, Piperidine-H), 1.65 (br, 6H, Piperidine-H); IR (KBr, cm^{-1}): ν_{max} 1691 (C=O), 1624 (C=N), 1611 (C=C); HRMS: *m*/*z* [M + Na]$^{+}$ calcd: 296.0832, found 296.0837.

(*Z*)-2-(Piperidin-1-yl)-5-(thiophen-2-ylmethylene)thiazol-4(5*H*)-one 9l

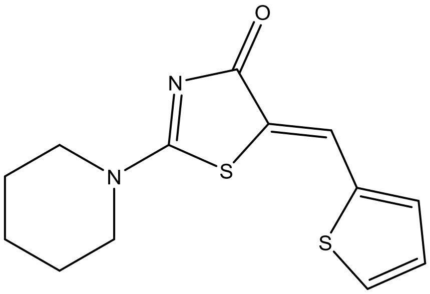

Yield 96%; greenish solid; mp = 203-205 °C; ^{1}H NMR (400 MHz, DMSO-*d6*) δ: 7.93 (d, 1H, J = 5.4 Hz, Ar-H), 7.87 (s, 1H, =C*H*), 7.59 (d, 1H, J = 3.5 Hz, Ar-H), 7.26-7.24 (dd, 1H, J = 1.4, 4.1 Hz, Ar-H), 3.96–3.88 (m, 2H, Piperidine-H), 3.66–3.63 (m, 2H, Piperidine-H), 1.62-1.65 ppm (m, 6H, Piperidine-H); IR (KBr, cm^{-1}): ν_{max} 1701 (C=O), 1609 (C=N), 1591 (C=C); HRMS: *m*/*z* [M]$^{+}$ calcd: 278.0548, found 278.0529.

(*Z*)-5-((1*H*-Indol-3-yl)methylene)-2-(piperidin-1-yl)thiazol-4(5*H*)-one 9m

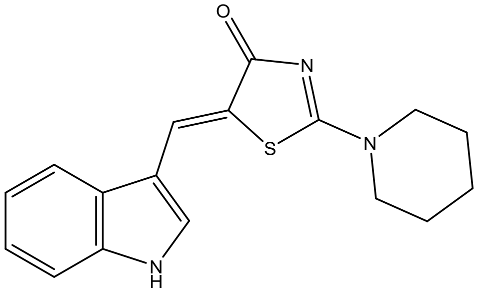

Yield 98%; yellow solid; mp = 244-246 °C; ^{1}H NMR (400 MHz, DMSO-*d6*) δ: 11.99 (s, 1H, NH), 7.89 (s, 1H, =C*H*), 7.85 (d, J = 8.7 Hz, 1H, Ar-H), 7.74 (s, 1H, Ar-H), 7.49 (d, J = 7.5 Hz,

1H, Ar-H), 7.19-7.11 (m, 2H, Ar-H), 3.88-3.78 (m, 4H, Piperidine-H), 1.78–1.70 ppm (m, 6H, Piperidine-H); IR (KBr, cm^{-1}): ν_{max} 1691 (C=O), 1612 (C=N), 1598 (C=C); HRMS: *m*/*z* $[M + H]^+$ calcd: 312.1167, found 312.1172.

SEM of TiO_2

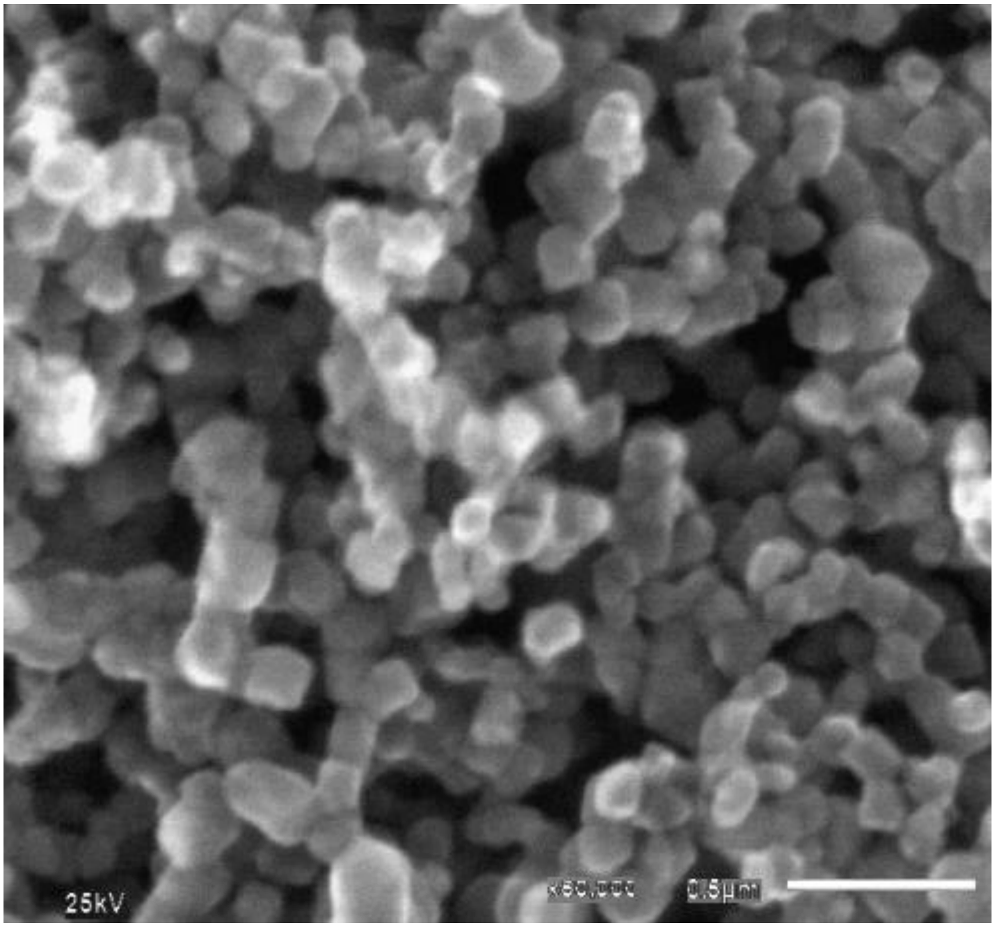

TEM of TiO_2

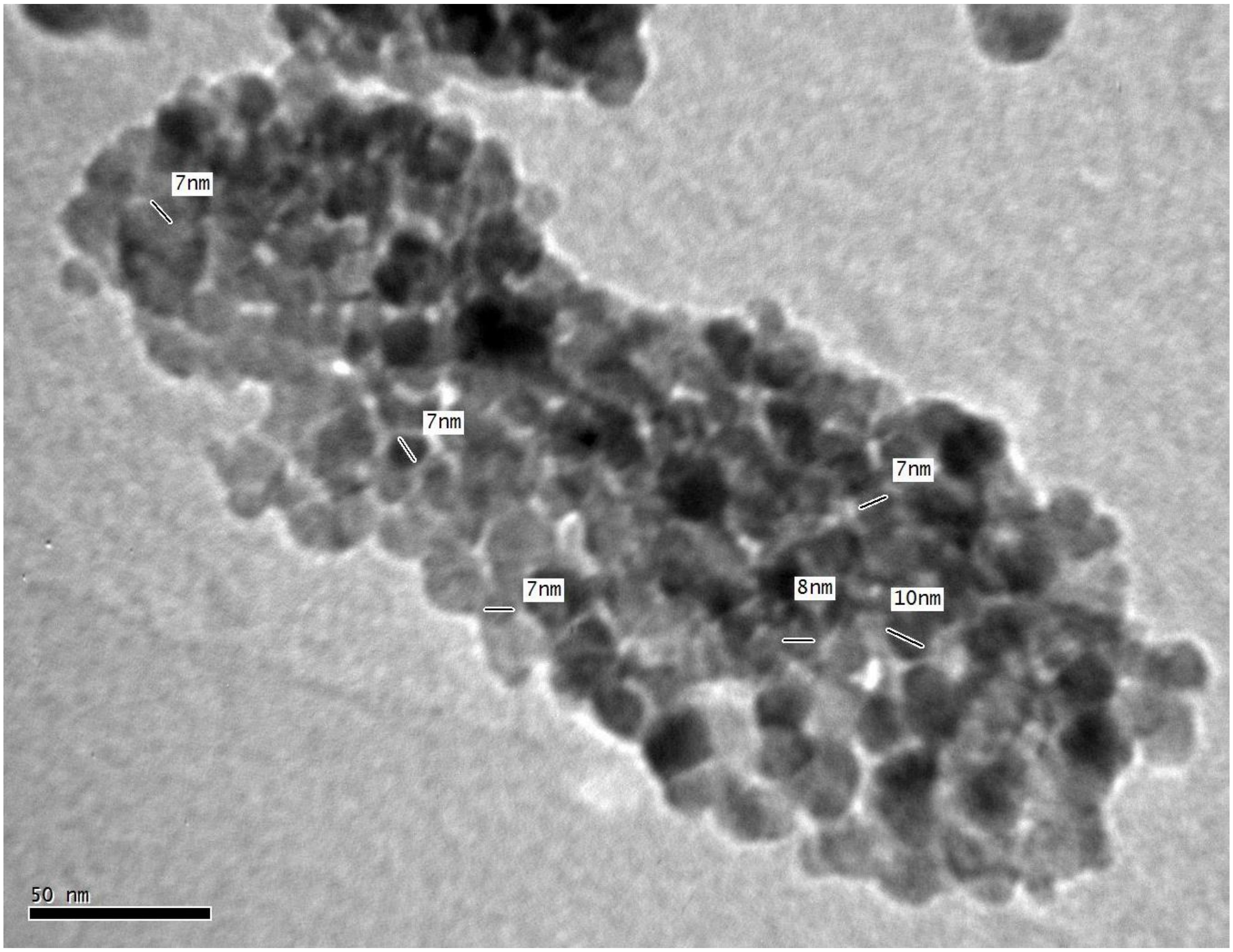

SEM of ZnO

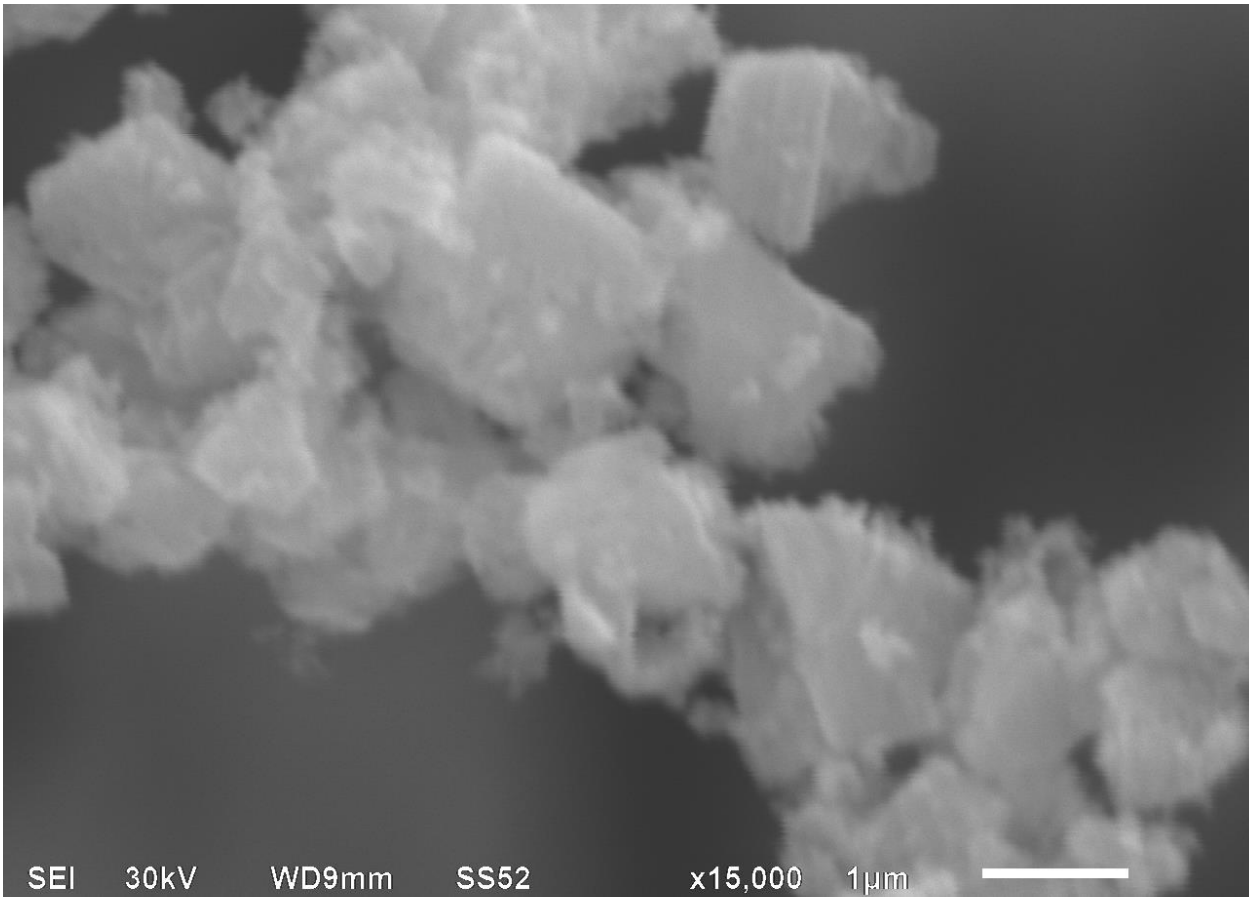

TEM of ZnO

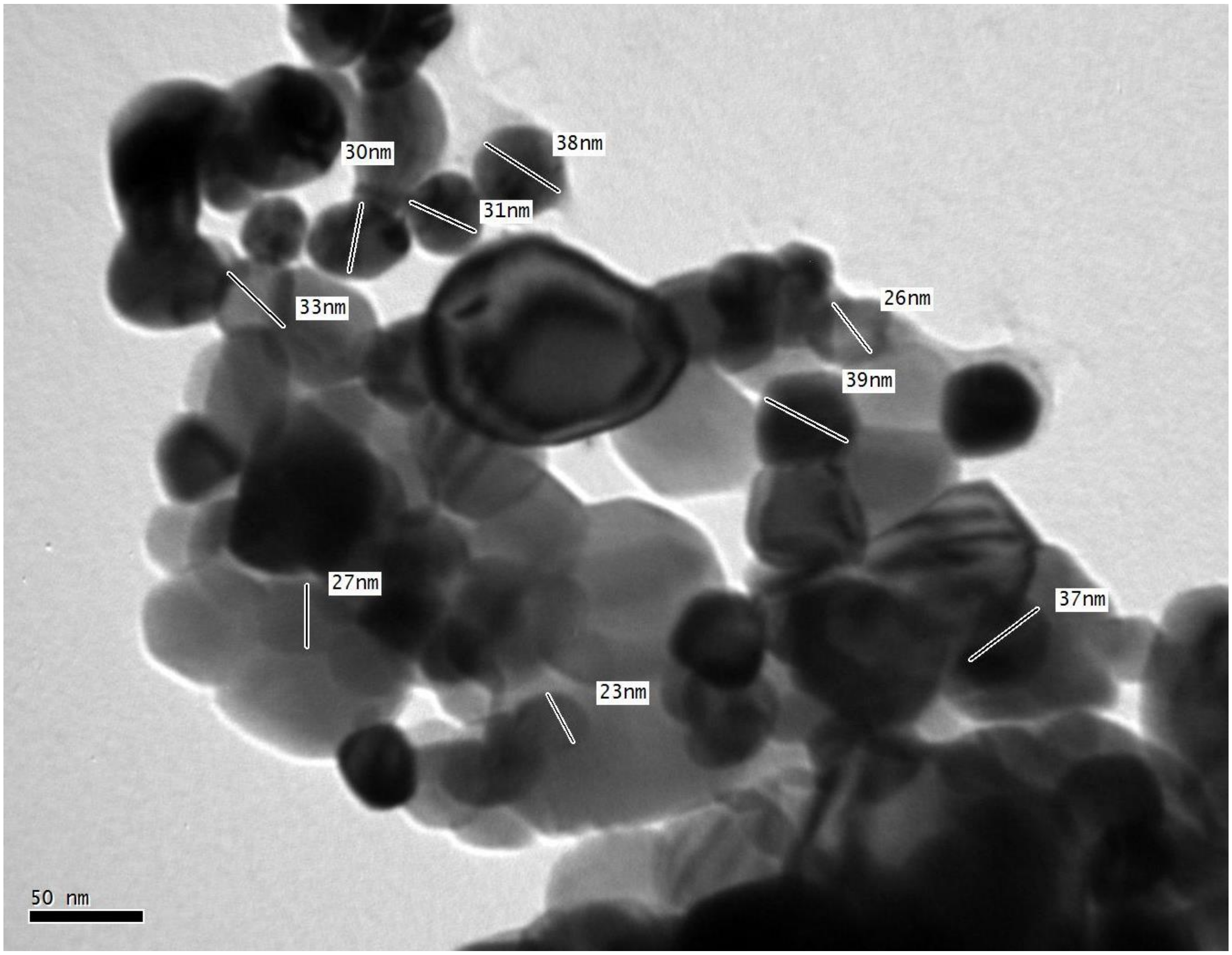

Green Metrics Calculations[1,2]

$$\textbf{\% Atomic Efficiency (AE)} = \frac{\text{Mol Wt. of desired product}}{\text{Mol Wt. of all reagents}} \times 100$$

$$\textbf{\% Carbon Efficiency (CE)} = \frac{\text{Mass of carbon in product}}{\text{Totall mass of carbon in the reactants}} \times 100$$

$$\textbf{Reaction Mass Efficiency (RME)} = \frac{\text{Mass of the isolated product}}{\text{Total mass of reactants used in the reaction}} \times 100$$

$$\textbf{\% Yield Economy (YE)} = \frac{\text{Reaction percent}}{\text{Time in min}} \times 100$$

$$\textbf{E-Factor (EF)} = \frac{\text{Mass of the total waste}}{\text{Mass of the crude product}}$$

$$\textbf{Process Mass Intensity (PMI)} = \frac{\text{Total mass used in process}}{\text{Mass of product}}$$

References

1. D. Curzons, D. J. C. Constable, D. N. Mortimer and V. L. Cunningham, *Green Chem.*, 2001, **3**, 1-6.
2. C. Jimenez-Gonzalez, D. J. C. Constable and C. S. Ponder, *Chem. Soc. Rev.*, 2012, **41**, 1485-1498.
